# Agave distribution and floral display influence foraging rates of an endangered pollinating bat and implications for conservation

**DOI:** 10.1002/ece3.11125

**Published:** 2024-03-15

**Authors:** Kristen M. Lear, Clinton T. Moore, Elizabeth G. King, Emma Gómez‐Ruiz, Jose Juan Flores Maldonado, Cuauhtemoc Ibarra Sanchez, Ana Castañeda Aguilera, Thomas J. Prebyl, Jeffrey Hepinstall‐Cymerman

**Affiliations:** ^1^ Bat Conservation International Austin Texas USA; ^2^ Warnell School of Forestry and Natural Resources University of Georgia Athens Georgia USA; ^3^ Integrative Conservation Program University of Georgia Athens Georgia USA; ^4^ Odum School of Ecology University of Georgia Athens Georgia USA; ^5^ Parque Ecológico Chipinque, A.B.P. San Pedro Garza García Nuevo León Mexico; ^6^ Especies, Sociedad y Hábitat, A.C. Apodaca Nuevo León Mexico

**Keywords:** agaves, bat conservation, endangered species, foraging analysis, Mexican long‐nosed bat (*Leptonycteris nivalis*), restoration

## Abstract

Wildlife conservation involves making management decisions with incomplete knowledge of ecological relationships. Efforts to augment foraging resources for the endangered Mexican long‐nosed bat (*Leptonycteris nivalis*) are progressing despite limited knowledge about the species' foraging behavior and requirements. This study aimed to understand *L. nivalis* responses to floral resource availability, focusing on individual agave‐ and local‐scale characteristics influencing visitation rates to flowering agaves. We observed bat visitation at 62 flowering agaves around two roosts in northeast Mexico on 46 nights in the summers of 2017 and 2018. We found visitation rate had positive relationships with two agave‐scale characteristics: the number of umbels with open flowers and the lower vertical position on the stalk of those umbels (i.e., earlier phenological stages of flowering). However, these factors exhibited strong negative interaction: with few umbels with open flowers, the position of flowering umbels had little effect on visitation rate, but when umbels with open flowers were abundant, visitation rate was more strongly related to the lower flowering umbel position. We also found relationships between visitation rate and two local‐scale characteristics: negative for the density of flowering conspecifics within 30 m of the focal agave and positive for the density of dead standing agave stalks within 30 m. Our findings suggest opportunities to augment foraging resources for *L. nivalis* in ways that are consistent with their foraging behavior, including: increasing the supply of simultaneously blooming flowers by planting agave species that tend to have more umbels with simultaneously open flowers; planting multiple species of agaves with different flowering times to increase the availability of agaves with open flowers on lower‐positioned umbels throughout the period when bats are present in the region; planting agaves in clusters; and keeping dead standing agave stalks on the landscape. Our study points to useful management strategies that can be implemented and monitored as part of an adaptive management approach to aid in conservation efforts.

## Graphical abstract

This study sought to fill knowledge gaps about the response of an endangered nectarivorous bat, the Mexican long‐nosed bat (*Leptonycteris nivalis*), to floral resource availability by addressing individual plant and local‐scale characteristics that influence bat visitation rates to flowering agaves. From nightly observations of bat foraging at 62 flowering agaves in northeast Mexico, we found that visitation rate had positive relationships with two agave‐scale characteristics (the number of umbels with open flowers and earlier phenological stages of plants) and one local‐scale characteristic (density of dead standing stalks within 30 m of the focal plant). We also found that visitation rate had a negative relationship with one local‐scale characteristic (density of flowering conspecifics within 30 m of the focal plant). Our findings suggest opportunities to augment foraging resources for *L. nivalis* in ways that are consistent with their foraging behavior, including increasing the supply of simultaneously blooming flowers by planting agave species that tend to have more umbels with simultaneously open flowers; planting multiple species of agaves with different flowering times to increase the availability of agaves with open flowers on lower‐positioned umbels throughout the period when bats are present in the region; planting agaves in clusters; and keeping dead standing agave stalks on the landscape. Our study points to useful management strategies that can be implemented and monitored as part of an adaptive management approach to aid in conservation efforts.

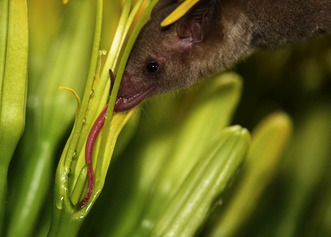

## 1 INTRODUCTION

Wildlife conservation involves making management decisions without complete knowledge. Uncertainty about the structure of biological and ecological relationships that drive the dynamics of complex systems is often high (Williams & Brown, 2016). The spatial and temporal scales of species' dynamics and processes, particularly for long‐distance migratory species, can also complicate the relationship between management actions and population‐level effects (Sutherland, 1998). These uncertainties limit the ability to understand how management interventions may affect the target system or organism (Nichols et al., 1995). However, a conservation urgency such as imminent species extinction may compel action while uncertainties are unresolved (Martin et al., 2012). The Mexican long‐nosed bat (*Leptonycteris nivalis*) exemplifies the progression of conservation activities, particularly efforts to augment foraging resources, despite limited knowledge about the species' foraging behavior and requirements.

**FIGURE 1:**
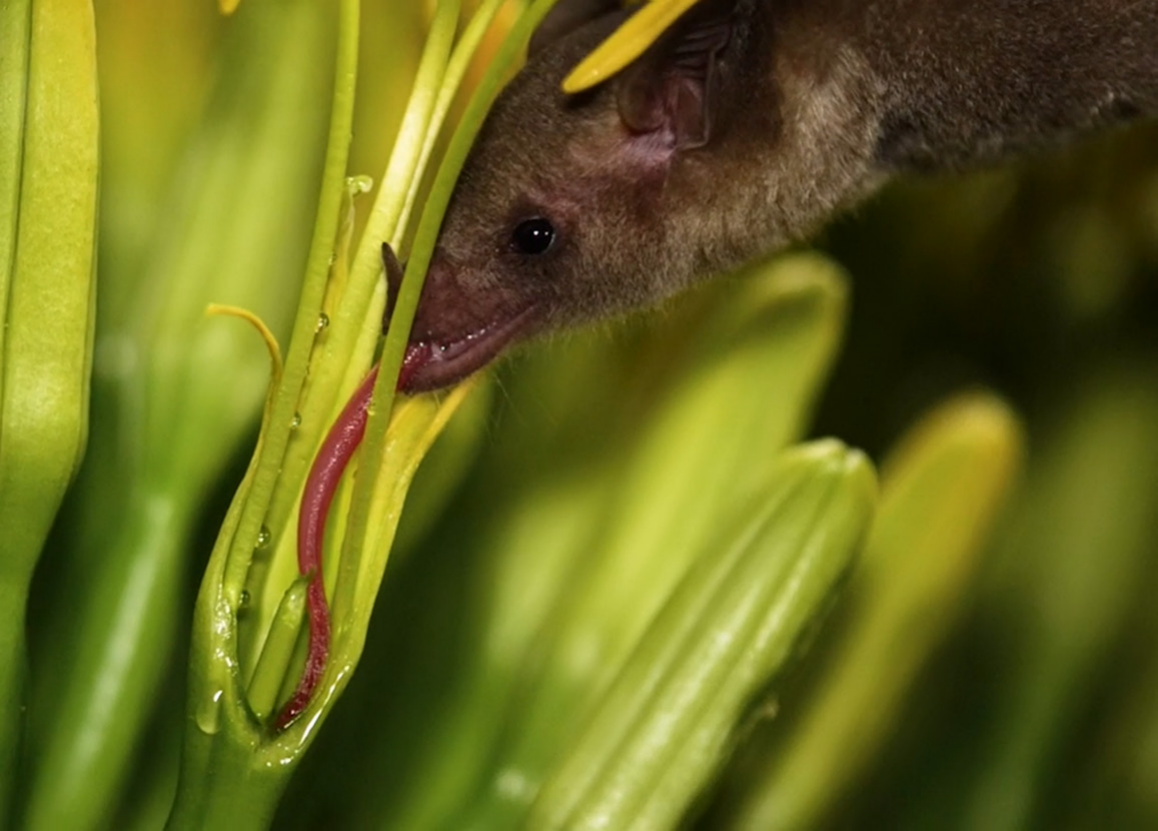
A Mexican long‐nosed bat (*Leptonycteris nivalis*) feeding on agave nectar. Photo credit: Horizonline Pictures, Bat Conservation International.

*Leptonycteris nivalis* is a migratory nectarivorous bat found in Mexico and the southwestern United States (Figure 1). It is listed as endangered in the United States (U.S. Fish and Wildlife Service, 1988) and by the IUCN Red List of Threatened Species (Arroyo‐Cabrales et al., 2008; Medellín, 2016) and is considered threatened in Mexico (NOM‐059‐SEMARNAT‐2010, SEMARNAT, 2010). During their annual migration between central Mexico and the southwestern United States, pregnant females follow a “nectar corridor” of flowering agave plants (*Agave* spp.) and cacti (Family Cactaceae) (Fleming et al., 1993; Gómez‐Ruiz & Lacher, 2017; Moreno‐Valdez et al., 2000). Because they rely primarily on the nectar and pollen of paniculate agaves in the northern portion of their range (England, 2012; Kuban, 1989; Moreno‐Valdez et al., 2004), species persistence likely depends on the timing and availability of flowering paniculate agaves and the connectivity of foraging areas across the migratory range (U.S. Fish and Wildlife Service, 2018, 2022) (Video 1). Therefore, protection and restoration of agave habitat is an identified recovery action for the species (U.S. Fish and Wildlife Service, 2022). Some conservation efforts currently focus on increasing the availability of flowering agaves around key roosting sites and along migratory pathways (e.g., Bat Conservation International, n.d.). Understanding the foraging behavior of this species is important for designing agave augmentation strategies to aid in its conservation.

**VIDEO 1:** A Mexican long‐nosed bat (*Leptonycteris nivalis*) feeding on the nectar of an agave flower. Photo credit: Horizonline Pictures, Bat Conservation International.

Previous research has documented several linkages between individual plant characteristics or local‐scale features and the feeding behavior of *L. nivalis* or the related Lesser long‐nosed bat (*Leptonycteris yerbabuenae*). Ober and Steidl (2004) found that visitation rates of *L. yerbabuenae* at flowering *Agave palmeri* in Arizona were positively related to a plant's floral display density but unrelated to the total abundance of umbels (flower clusters). In western Texas, *L. nivalis* preferentially visited *Agave havardiana* branches with more open flowers (Kuban, 1989). In addition, Howell (1979) observed that foraging *L. yerbabuenae* responded positively to experimental increases in nectar volume of *A. palmeri* flowers. Also, agave flowers open sequentially up the stalk, so that only a few umbels are ever open with available nectar. Ober and Steidl (2004) found an association between visitation rates of *L. yerbabuenae* and the phenology of *A. palmeri*, with the visitation rate being maximized when umbels with open flowers occurred in the middle of the stalk.

Bat visitation rates may depend on foraging resource cues at different scales. Although previous studies have found no links between *Leptonycteris* visitation rates to focal plants and the density of nearby flowering plants (Horner et al., 1998; Ober & Steidl, 2004), bats may exhibit landscape‐scale habitat selection and may concentrate foraging in areas of higher food abundance. Ober et al. (2005) found greater density of flowering agaves in home ranges of adult *L. yerbabuenae* than in the landscape at large, and Bogan et al. (2017) suggest that *L. nivalis* in New Mexico consistently forage in areas with higher densities of flowering agaves.

Foraging bats may also cue in on areas with evidence of high food abundance in the past. Ober et al. (2005) found that *L. yerbabuenae* selected areas with a high density of dead standing stalks, an indicator of abundant food in previous years. Because these bats may use both olfactory and visual cues to guide foraging (Howell, 1979), the density of agave stalks in all phenological stages may be important to consider in foraging studies.

Few studies have examined the foraging activity of *L. nivalis* in northeast Mexico (Gómez‐Ruiz, 2015; Gómez‐Ruiz & Lacher, 2017; Moreno‐Valdez et al., 2000, 2004); thus, factors that influence feeding rates on agaves in the northern portion of the species' range are largely unknown. Females migrate to northeast Mexico to birth and rear pups in at least two caves, and this area is also the focus of conservation efforts to increase foraging resources (flowering agaves). We sought to fill knowledge gaps about the bats' responses to floral resource availability, specifically questioning what individual agave and local‐scale characteristics influenced bat visitation rates to flowering agaves in northeast Mexico. We studied relationships between foraging behavior and agave‐scale and local‐scale variables by observing bat visitation rates at flowering agaves in two regions around two main roosting caves in the Mexican states of Nuevo León and Coahuila. In line with the findings of earlier studies reviewed above, we predicted that we would find positive relationships between bat visitation and the number of umbels with open flowers, the number of nearby flowering conspecifics, and the number of nearby dead standing agave stalks. We also expected to find a relationship between visitation rate and the relative vertical position of umbels with open flowers.

## 2 MATERIALS AND METHODS

### 2.1 Study sites

**FIGURE 2:**
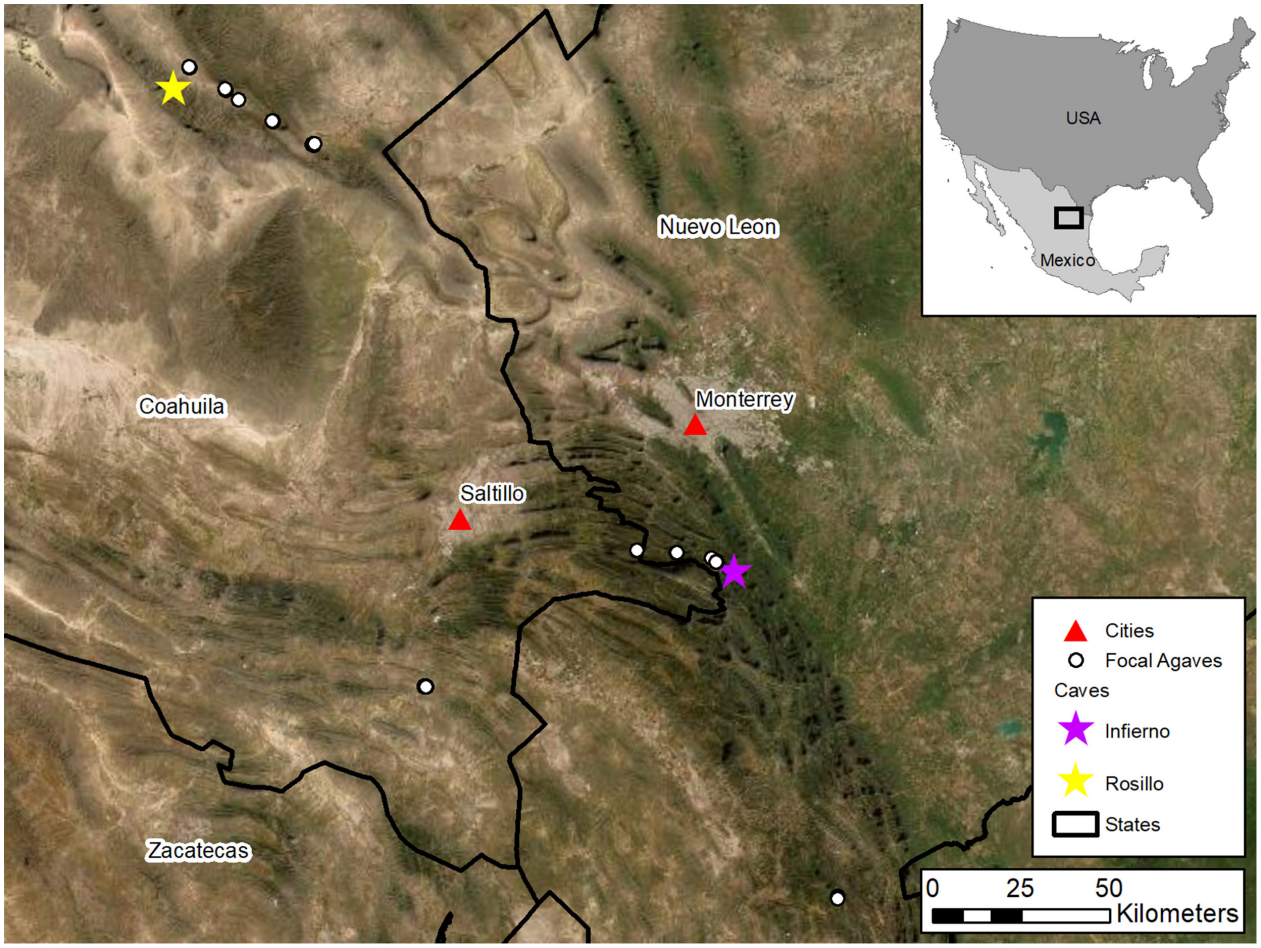
Northeast Mexico study area, with two roosting caves indicated: Infierno Cave in Nuevo León (purple star) and Rosillo Cave in Coahuila (yellow star). Focal agaves are shown as white dots (note that due to the large spatial extent, many focal agaves overlap in the image). The focal agaves in the state of Coahuila belong to the “Rosillo region” and those in Nuevo León belong to the “Infierno region” in our analysis. Aerial imagery from Maxar Vivid Imagery (10/17/2016); State boundaries from INEGI (2010).

We conducted our study in the northeast Mexican states of Nuevo León and Coahuila (Figure 2), in the northern portion of the migratory range of *L. nivalis*. This is the natal region for the species and contains two important roosting caves: Infierno Cave, a confirmed maternity cave (Parque Nacional Cumbres de Monterrey, Nuevo León) and Rosillo Cave, a suspected maternity cave (Área de Protección de los Recursos Naturales CADNR004 Cuenca Don Martín, Coahuila). Infierno Cave (approximately 1620 m ASL) is located among pine‐oak forests in the Sierra Madre Oriental Mountains. Rosillo Cave (approximately 1780 m ASL) is located in desert scrub in the Chihuahuan Desert. Agaves are the sole known source of nectar for *L. nivalis* here (England, 2012; Kuban, 1989; Moreno‐Valdez et al., 2004).

Agaves are long‐lived succulent plants, with approximately 200 species in the genus *Agave*. They are native to North America and show the highest species diversity in Mexico (166 species) (García‐Mendoza et al., 2017; Gentry, 1982). Agaves only flower once in their life, then die. After years of maturation, agaves produce a large flowering stalk with thousands of nectar‐rich flowers. In paniculate agave species (the only known nectar source for *L. nivalis* in northeast Mexico), flowers are arranged on lateral branches in groups called umbels. Using species descriptions in Gentry (1982), we identified three paniculate agave species in our study sites and surrounding communities near Rosillo Cave (Rosillo region) and five paniculate species in our study sites and surrounding communities near Infierno Cave (Infierno region) (Table S1). *Agave lechuguilla* (a spicate species with an unbranched flowering stalk) is also common in many of our study sites but is not known to be a food source for *L. nivalis* in northeast Mexico. Some species tend to flower earlier in the year than others, but even within species, the flowering of different individuals in a site can span several months.

### 2.2 Sampling design and data collection

We monitored bat foraging rates, measured as visits to a focal agave per bat per hour, at 12 sites (six per study region), each approximately 3 km^2^, with relatively abundant but variable densities of agaves. We selected sites based on the availability of flowering agaves and accessibility. During each site visit, we identified 2–4 flowering agaves (with open and active nectar‐producing flowers), each serving as the focal center of a 30‐m radius area containing varying densities of flowering agaves. We monitored the focal agaves at night, with the sampling effort designed to avoid measuring plants with overlapping radii on the same night (achieved in all but two instances).

We simultaneously monitored 2–4 focal agaves for bouts of 1–3 consecutive nights. These concurrently monitored focal agaves are termed “clusters.” On nights with camera equipment difficulties, we reduced the number of focal agaves monitored. On subsequent bouts, we identified a new set of focal agaves (i.e., a new cluster) to monitor. Since each cluster was observed for only one bout, cluster was included as a factor in our statistical models to account for seasonal variability in bat abundance, foraging behavior, or agave floral abundance at the sites (see Figures S1 and S2 for aerial photos of a representative cluster and a representative site, respectively).

We conducted agave surveys and measured the following characteristics of each focal agave (agave‐scale variables) to use as explanatory variables of foraging rate in our statistical models (Table S2): (1) total number of umbels; (2) number of umbels containing open flowers; and (3) relative vertical position of the umbels containing open flowers. Over the course of flowering, agave flowers open sequentially from the bottom of the stalk upward, with only a few umbels having open flowers at any time. The relative position of umbels with open flowers along the stalk, expressed as a value between 0 and 1, reflects the phenology of the individual agave. Lower‐positioned flowering umbels (relative position value near 0) indicate an early phenological status of the plant, and higher‐positioned flowering umbels (position values near 1) indicate a late phenological status. Agave species was not included as an explanatory variable in our statistical models given the high levels of phenotypic variation and overlapping traits between species and our inability to visually distinguish between species reliably. We measured five local‐scale variables, determined within the 30‐m radius of each focal agave (following Horner et al., 1998): (1) density of young agave stalks (containing umbels with unopened flowers); (2) density of agave stalks with any open flowers; (3) density of agave stalks with senescent flowers that no longer have nectar available; (4) density of dead standing agave stalks that had flowered in previous years; and (5) density of all agave stalks of any type. At each focal agave, we also recorded elevation and estimated the slope by visually estimating the angle formed (to the nearest 5 degrees) between the run over 10 m surrounding the focal agave and an idealized flat (horizontal) surface.

We recorded foraging activity with one digital video camera placed 8–15 m away (Sony FDR‐AX33 and FDR‐AX53, using the Nightshot feature). We used two infrared lamps (IR6 Lamps, Wildlife Engineering, Tucson, Arizona, USA) per agave to provide clear illumination of all umbels. We typically recorded each cluster of focal agaves for approximately 6 h per night, but inclement weather curtailed monitoring on some nights. We recorded the monitoring effort to the nearest minute.

We counted visits of nectarivorous bats to each focal agave by viewing video recordings using VLC Media Player (version 2.0.1), slowing down playback as necessary. *L. nivalis* accounted for most visits, but we occasionally observed the sympatric Mexican long‐tongued bat (*Choeronycteris mexicana*). We observed but excluded from our counts insectivorous bats, mainly Pallid bats (*Antrozous pallidus*), that have been documented in other foraging studies to feed from blooming agaves and cacti (e.g. Frick et al., 2009; Herrera et al., 1993; Hinman, 2003; Jaquish & Ammerman, 2021; Kuban, 1989). We distinguished nectarivorous bats from others based on flight traits (more direct, not as lilting), foraging behavior (flower visits <2 s), and physical characteristics (V‐shaped tails in both species (Hoffmeister, 1986); a distinguishable long nose in *C. mexicana*). As cameras recorded, we also scanned the field of view periodically with night vision binoculars to count bats for later corroboration with video images.

We defined a visit as a flight by a bat flying up to or beside the umbel, followed by a pause, then a swoop down and away (Kuban, 1989). Many visits by *L. nivalis* included contact with the flowers, as evidenced by the physical movement of the umbel. We defined a flyby as any pass in the video frame by a nectarivorous bat that did not entail a visit.

### 2.3 Statistical analyses and model selection

The response variable for analysis was the count of visits to a focal agave, with an offset adjustment to standardize for time monitored and the number of bats foraging at the agave. Our use of a *per‐bat visitation rate* contrasts with previous studies of *Leptonycteris* foraging (Horner et al., 1998; Ober & Steidl, 2004) that did not account for the number of bats foraging at the focal plants. Because *Leptonycteris* bats often forage in small groups and feed in sequence at numerous flowering plants in a site (Howell, 1979), our statistical models used an offset term of log(t*b + 1), where *t* was the duration (minutes) that the focal agave was monitored, and *b* was the greatest number of bats observed at any focal agave, either as a visit or as a flyby, at any moment over the night (as determined by the maximum number of bats seen in the field of view of any of the cameras on a given night, supported by periodic night vision binocular scans of the study site by the lead author who identified nectarivorous bats by their flight traits, foraging behavior, and physical characteristics as described previously). By standardizing the response variable to a per‐bat measure, our models account for the linear effect that changes in overall bat abundance are expected to have on total visits per plant.

To examine the individual agave‐ and local‐scale factors that influenced per‐bat visitation rate to focal agaves, we used a generalized linear mixed model (GLMM) with the *glmmTMB* function (R package *glmmTMB* v1.0.1; Brooks et al., 2017a, 2017b). Counts of zero arose in our data by chance because either bats were not present on certain nights or bats at the focal agaves were not captured by the cameras. Therefore, we chose a mixture model (a zero‐inflated Poisson model with a log link) to probabilistically distinguish expected (bats never expected to visit) from random (bats not visiting by chance) zeroes (He et al., 2014). We used the cluster of 2–4 agaves monitored on a night as a random effect for the zero‐inflation component of the model. Since each cluster was monitored only once during the season, this random effect also accounts for seasonal or year‐to‐year variation in bat abundance or behavior.

We checked for collinearity between all pairs of explanatory variables using Pearson correlation coefficients. We found elevation and slope moderately correlated (*r* = .67), so we removed elevation and retained slope in our models for its potential role in the selection of management sites for conservation activities. We then created an initial global model with the following terms: linear fixed effects for all agave‐scale (three) and local‐scale (five) variables; an interaction between the number of umbels with open flowers and the relative vertical position of umbels with open flowers; and a quadratic term for relative position. We included the last term to assess whether the visitation rate was highest when the umbels with open flowers occurred in the middle of the flowering stalk, as Ober and Steidl (2004) had found in their study of *L. yerbabuenae* foraging. We included a three‐level random effect to capture variation among focal agaves nested within clusters nested within sites. We scaled continuous variable fixed effects using a *z*‐score transformation to facilitate comparison between model parameters (Schielzeth, 2010). From the initial model, we removed two local‐scale variables, the density of all agave stalks and the density of young agave stalks, which were identified as problematic because of collinearity or model convergence issues. From the remaining variables, we prepared a set of 21 a priori GLMM models containing various combinations of the selected variables as fixed effects as well as the nested random effect in all models.

We used an information‐theoretic approach (Burnham & Anderson, 1998, 2002) to select among competing models and evaluate the relative influence of agave‐ and local‐scale variables on per‐bat visitation rates for flowering agaves. We conducted model selection using the small‐sample corrected Akaike Information Criterion (AIC_c_, Akaike, 1973) to rank models. We used a significance threshold of *p* < .05 to make inferences within specific models.

We accounted for model selection uncertainty through model averaging and obtained robust parameter estimates and predictions with higher precision (Dormann et al., 2018; Grueber et al., 2011). We averaged over the smallest set of models that accounted for ≥95% of the cumulative AIC_c_ weight (95% confidence set, Burnham & Anderson, 2002; Symonds & Moussalli, 2011). We used the zero method for model averaging (i.e., we substituted zero for a parameter estimate and its associated standard error where the given parameter was absent; Burnham & Anderson, 2002), which is recommended when the aim of the study is to determine which factors have the strongest effect on the response variable (Grueber et al., 2011; Nakagawa & Freckleton, 2010). We created graphs of the predicted per‐bat visitation rate for the range of observed values of predictor variables of interest on the x‐axis, holding all other predictor variables constant at their means. We obtained model‐averaged parameter estimates and predictions using the *model.avg* and *predict* functions, respectively, in the R package *MuMIn* v1.43.17 (Barton, 2020). We used R version 3.6.3 (R Core Team, 2020) for all statistical analyses. The data that support the findings of this study are openly available in the Knowledge Network for Biocomplexity at https://doi.org/10.5063/F1QJ7FST (Lear, 2023).

## 3 RESULTS

### 3.1 Visitation trends in the study regions

Between May 26–July 31, 2017 and April 28–July 28, 2018, we monitored foraging over 688 h on 46 nights (297 h on 23 nights for the 6 Infierno sites and 391 h on 23 nights for the 6 Rosillo sites). We monitored a total of 62 flowering agaves (28 at Infierno sites and 34 at Rosillo sites), including *Agave americana*, *Agave asperrima*, *Agave salmiana*, *Agave gentryi*, and *Agave montana*. We monitored 1–4 clusters of focal agaves per site. Sites represented a range of elevation and slope (Table S3; see Table S4 for site‐level descriptive statistics).

Across both years and study regions, we recorded 26,128 nectarivorous bat visits. By region, we tallied over seven times as many total bat visits (23,078) at the 28 agaves in the Infierno region than at the 34 agaves in the Rosillo region (3050).

Total visits per hour (i.e., not adjusted for numbers of bats observed) across both regions ranged from 0 to 571.6 and averaged 37.0 visits per hour (Infierno average 74.7 visits per hour, range 0–571.6; Rosillo average 7.44 visits per hour, range 0–128.9; Table S3). The maximum number of bats observed at any of the focal agaves on a single night we monitored ranged from 0 to 6 bats at Infierno sites and 0–4 bats at Rosillo sites (Table S3). Accounting for the number of bats present and foraging at the focal agaves, the average per‐bat visitation rate across the study was 9.6 visits per bat per hour (18.6 visits per bat per hour at Infierno agaves and 2.6 visits per bat per hour at Rosillo agaves; Table S3). While we do not have colony size numbers for Infierno and Rosillo Caves during the study period, our use of per‐bat visitation rate and inclusion of clusters as a random effect account for fluctuations in overall bat abundance.

### 3.2 Variables influencing per‐bat visitation rate to focal agaves

**FIGURE 3:**
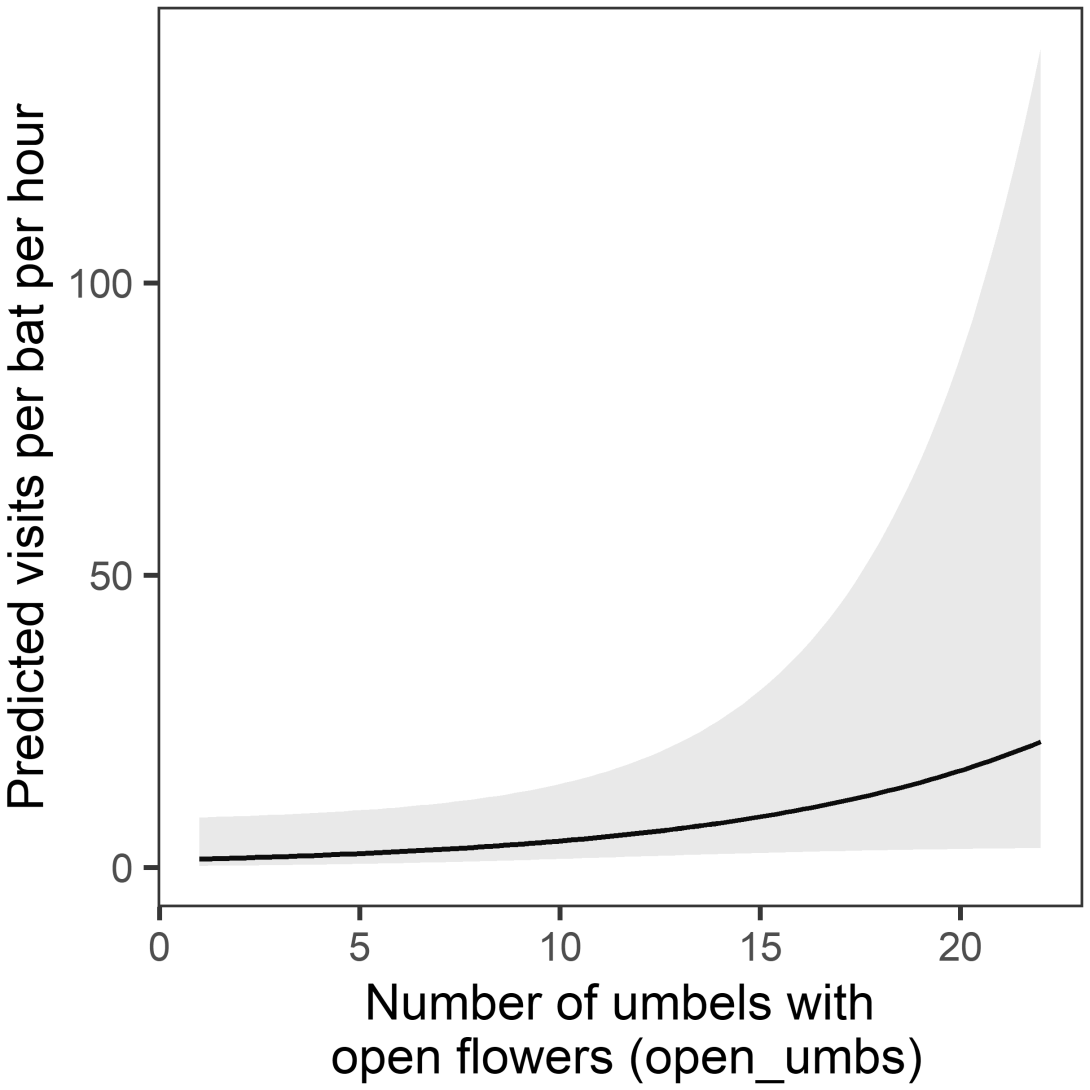
Model‐averaged prediction (95% confidence interval shaded) of the effect of the number of umbels with open flowers (*open_umbs*) on per‐bat visitation rate at focal agaves.

**FIGURE 4:**
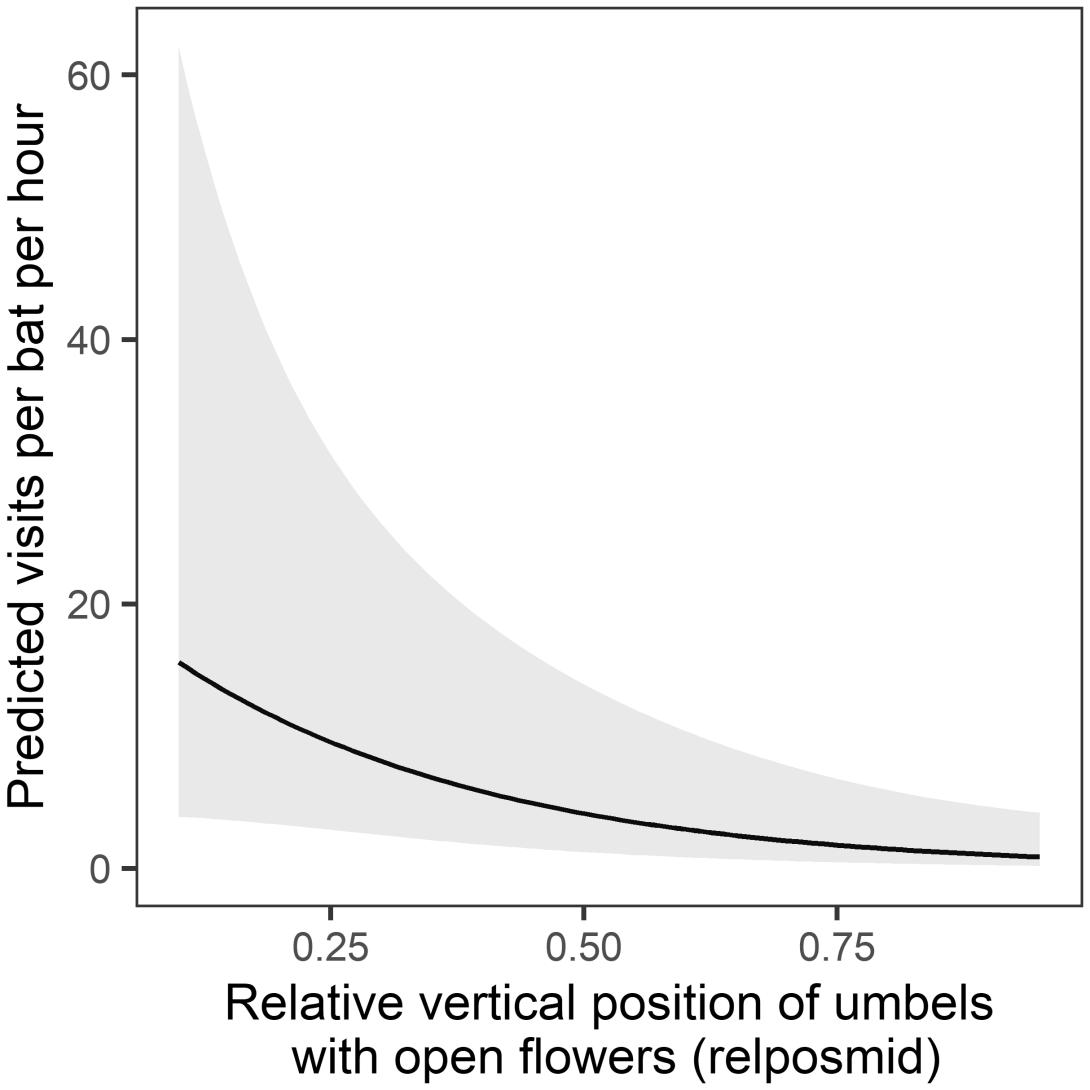
Model‐averaged prediction (95% confidence interval shaded) of the effect of relative umbel position (*relposmid*) on per‐bat visitation rate at focal agaves. Low values of *relposmid* mean that the umbels with open flowers are the bottom‐most umbels (indicating earlier phenological status), while high values mean that the umbels with open flowers are at the top of the flowering stalk (indicating later phenological status).

We report results for the 13 models in the 95% confidence model set (Table 1). Standardized parameter estimates for each variable are presented in Table S5, and standardized and unstandardized model‐averaged estimates are presented in Table S6. We found two agave‐scale variables significant in predicting per‐bat visitation rate (Table S5). The number of umbels with open flowers on a plant and their relative position on the stalk were present as predictors in all the models and were significant in 5 and 12 of the 13 models, respectively. Per‐bat visitation rate varied positively with number of umbels with open flowers (model‐averaged estimate: *β* = 0.539 ± 0.294 SE, Table S6, Figure 3). Visitation rate varied negatively with the relative vertical position of umbels with open flowers, with greater visitation rate occurring at plants with lower‐positioned umbels with open flowers (model‐averaged estimate: *β* = −0.914 ± 0.266 SE, Table S6, Figure 4).

**TABLE 1:** Candidate model ranking is based on AIC_c_.

Model	Model structure	*K*	AIC_c_	LL	ΔAIC_c_	*ω*_*i*_	Cumulative *ω*	*R* ^2^
1	slope + tot_umbs + open_umbs + relposmid + dens_open + dens_fuzz + dens_dry + open_umbs*relposmid	**14**	**3371.52**	**−1669.85**	**0.00**	**0.361**	**0.361**	**.38**
2	reg + slope + tot_umbs + open_umbs + relposmid + dens_open + dens_fuzz + dens_dry + open_umbs*relposmid	15	3373.89	−1669.74	2.37	0.111	0.472	.36
3	slope + tot_umbs + open_umbs + relposmid + dens_open + dens_fuzz + dens_dry + open_umbs*relposmid + relposmid^2^	15	3374.10	−1669.85	2.58	0.100	0.571	.38
4	slope + tot_umbs + open_umbs + relposmid + dens_open + dens_fuzz + dens_dry	13	3374.18	−1672.45	2.66	0.096	0.667	.37
5	reg + open_umbs + relposmid	9	3374.95	−1677.69	3.43	0.065	0.732	.36
6	open_umbs + relposmid	8	3375.37	−1679.07	3.85	0.053	0.785	.34
7	slope + tot_umbs + open_umbs + relposmid + dens_open + dens_fuzz + dens_dry + relposmid^2^	14	3376.39	−1672.28	4.86	0.032	0.816	.37
8	reg + slope + tot_umbs + open_umbs + relposmid + dens_open + dens_fuzz + dens_dry + open_umbs*relposmid + relposmid^2^	16	3376.52	−1669.74	5.00	0.030	0.846	.36
9	reg + slope + tot_umbs + open_umbs + relposmid + dens_open + dens_fuzz + dens_dry	14	3376.65	−1672.42	5.13	0.028	0.874	.38
10	reg + open_umbs + relposmid + open_umbs*relposmid	10	3376.76	−1677.42	5.24	0.026	0.900	.35
11	reg + open_umbs + relposmid + relposmid^2^	10	3377.12	−1677.59	5.59	0.022	0.922	.37
12	open_umbs + relposmid + open_umbs*relposmid	9	3377.28	−1678.86	5.75	0.020	0.942	.33
13	open_umbs + relposmid + relposmid^2^	9	3377.57	−1679.00	6.05	0.018	0.960	.35
14	reg + slope + tot_umbs + open_umbs + relposmid + dens_open + dens_fuzz + dens_dry + relposmid^2^	15	3378.88	−1672.24	7.36	0.009	0.969	.38
15	reg + open_umbs + relposmid + open_umbs*relposmid + relposmid^2^	11	3379.07	−1677.37	7.55	0.008	0.977	.35
16	open_umbs + relposmid + open_umbs*relposmid + relposmid^2^	10	3379.59	−1678.83	8.07	0.006	0.984	.33
17	slope + dens_open + dens_fuzz + dens_dry	10	3380.58	−1679.33	9.06	0.004	0.988	.33
18	tot_umbs + open_umbs + relposmid + open_umbs*relposmid + relposmid^2^	11	3380.78	−1678.22	9.26	0.004	0.991	.32
19	reg + slope + dens_open + dens_fuzz + dens_dry	11	3380.83	−1678.25	9.31	0.003	0.995	.35
20	reg + tot_umbs + open_umbs + relposmid + open_umbs*relposmid + relposmid^2^	12	3380.87	−1677.04	9.34	0.003	0.998	.35
21	Null	6	3381.94	−1684.61	10.42	0.002	1.000	.31

*Note*: All models included a three‐level nested random effect of focal agave nested within clusters nested within sites. The most parsimonious model (i.e. the model with ΔAIC_c_ ≤ 2) is shown in bold, and the number of parameters (*K*), AIC_c_ value, log‐likelihood (LL), difference in AIC_c_ values (ΔAIC_c_), model weight (*ω*_*i*_), and cumulative model weight (cumulative *ω*) are given. The model fit was characterized using the conditional *R* ^2^. Description of predictor variables: slope (slope as estimated at each focal agave); tot_umbs (total number of umbels on the agave); open_umbs (number of umbels containing open flowers on the agave); relposmid (relative vertical position along the stalk of umbels containing open flowers); dens_open (number of agave stalks with any umbels with open flowers within 30 m of the focal agave); den_fuzz (number of agave stalks with senescent flowers within 30 m of the focal agave); dens_dry (number of dead standing agave stalks within 30 m of the focal agave); open_umbs*relposmid (interaction between the number of umbels containing open flowers and the relative vertical position of the umbels containing open flowers); reg (region: Infierno or Rosillo).

**FIGURE 5:**
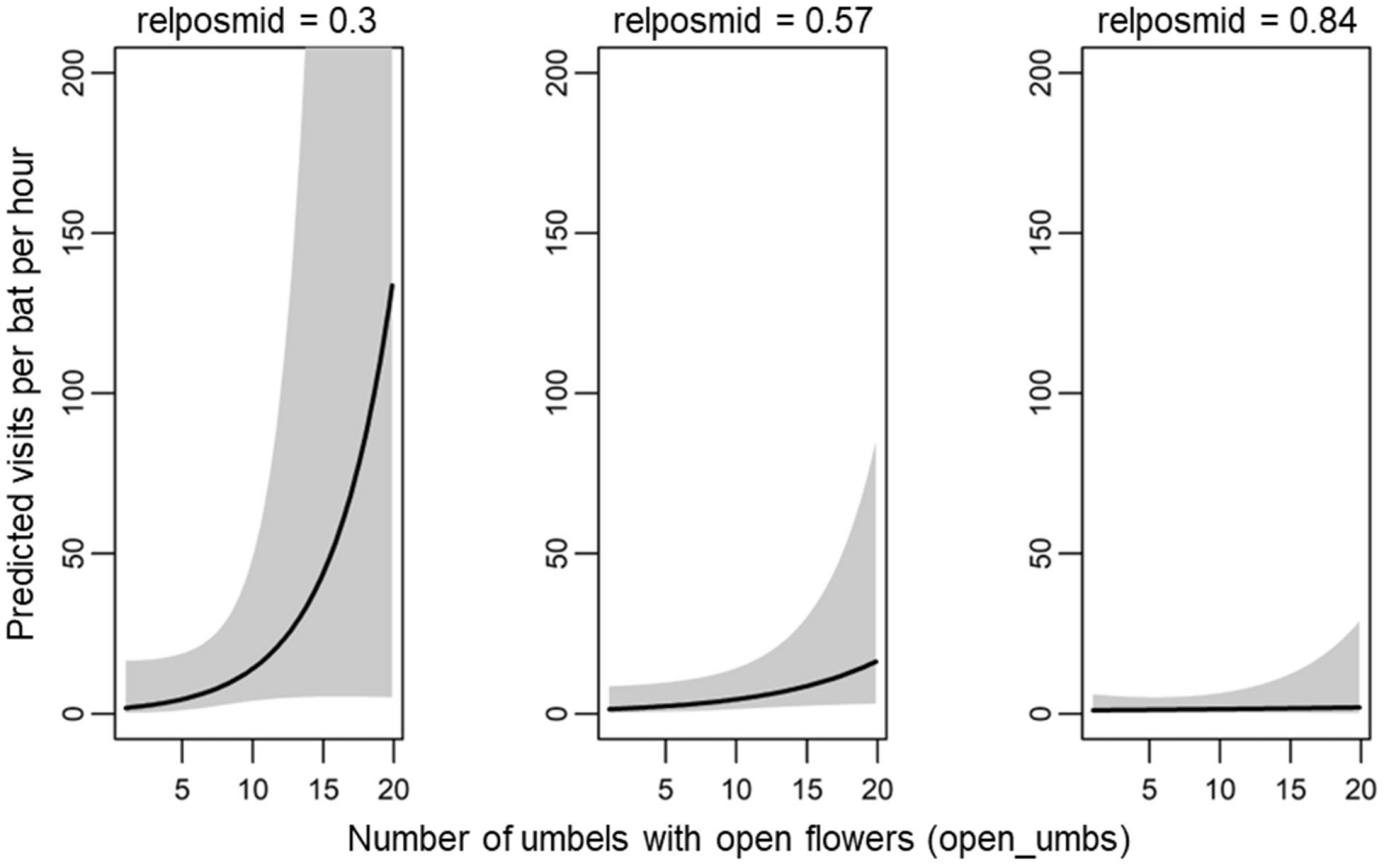
Model‐averaged prediction (95% confidence intervals shaded) of the effect of the interaction between the number of umbels with open flowers (*open_umbs*) and relative vertical position of those umbels (*relposmid*) on per‐bat visitation rate at focal agaves, shown at values of *relposmid* held fixed at 0.3, 0.57, and 0.84 (mean ± 1 SD), reflecting open flowers toward the bottom, the middle, and the top of the stalk's umbels, respectively. We limited the *y‐*axis to approximately 200 for visualization.

**FIGURE 6:**
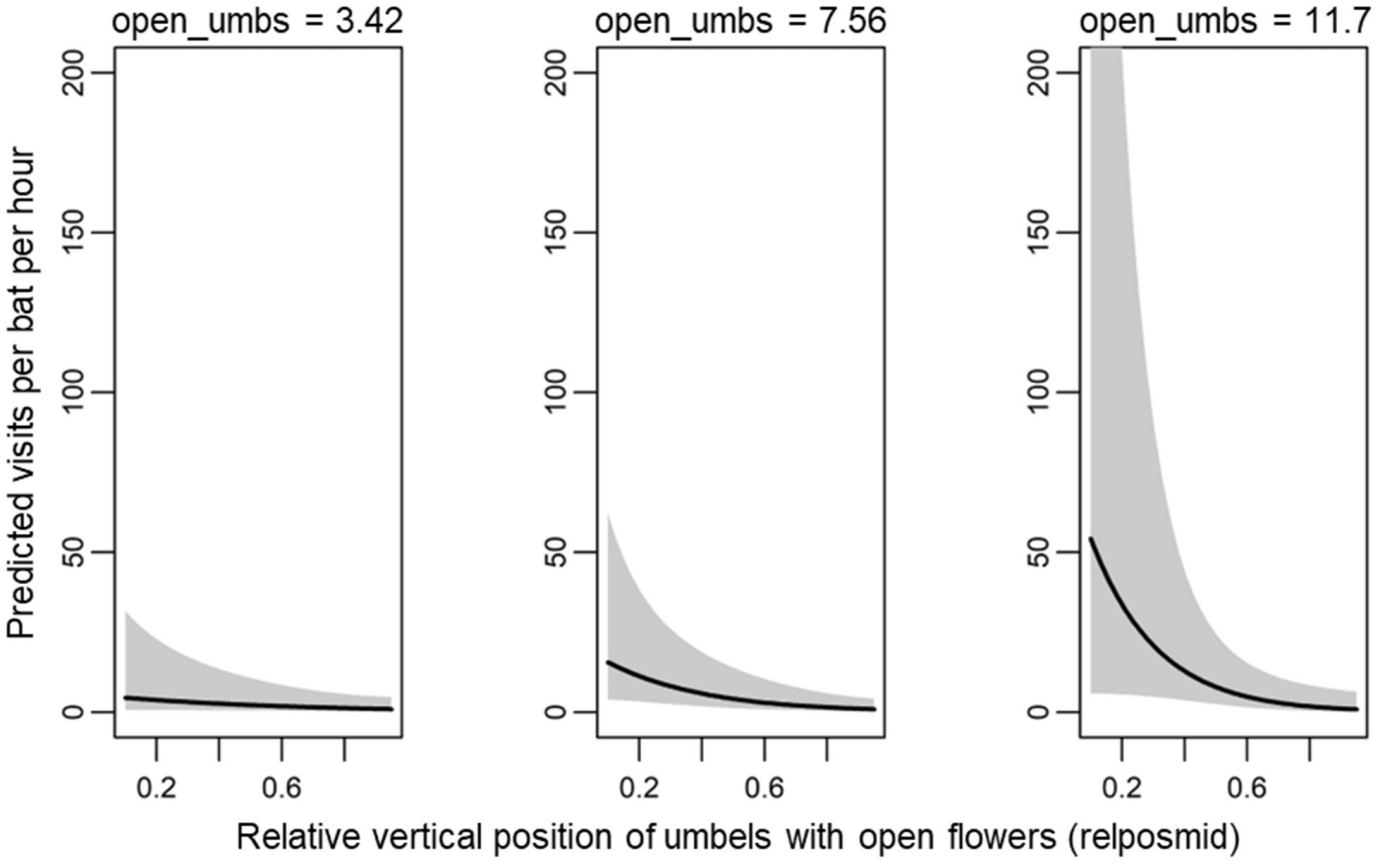
Model‐averaged prediction (95% confidence intervals shaded) of the effect of the interaction between the number of umbels with open flowers (*open_umbs*) and the relative vertical position of those umbels (*relposmid*) on the per‐bat visitation rate at focal agaves, shown at the values of *open_umbs* held fixed at 3.42, 7.56, and 11.7 (mean ± 1 SD). We limited the *y*‐axis to approximately 200 for visualization.

The interaction between the number of umbels with open flowers and their relative position was present in six of the 13 models and significant in four of those (Table S5). The regression coefficient was negative (model‐averaged estimate: *β* = −0.405 ± 0.373 SE, Table S6), indicating no effect of number of umbels with open flowers on per‐bat visitation rate when flowers were located at the top of the stalk (late flowering stage) and a strong positive effect of number of umbels with open flowers when these umbels were at the bottom of the stalk (early flowering stage; Figure 5). With few umbels with open flowers, their relative position had little effect on visitation rate, but when there were many umbels with open flowers, visitation rate was more strongly related to the lower umbel position (Figure 6).

**FIGURE 7:**
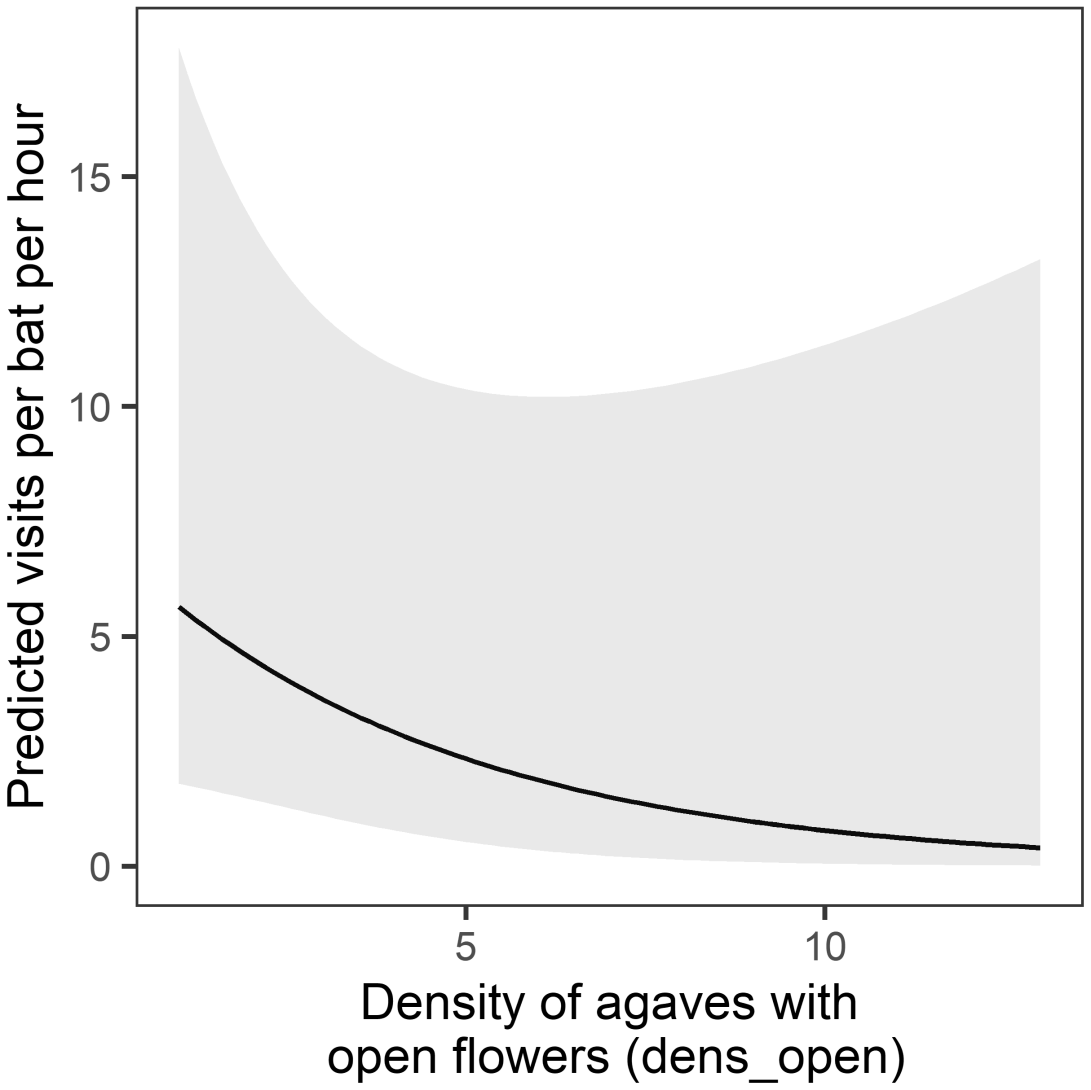
Model‐averaged prediction (95% confidence interval shaded) of the effect of number of agaves with open flowers (*dens_open*) within 30 m of the focal agave on per‐bat visitation rate at focal agaves.

**FIGURE 8:**
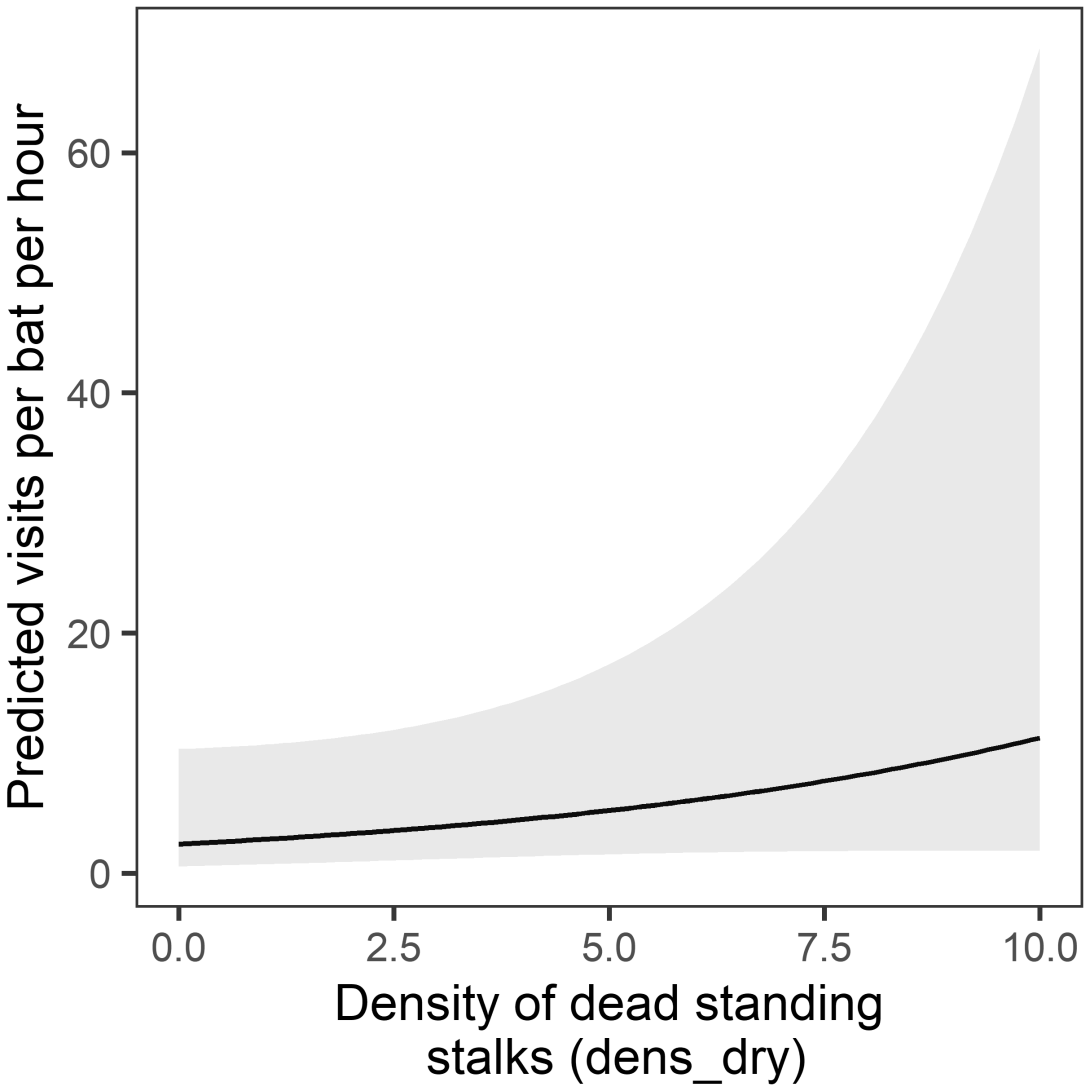
Model‐averaged prediction (95% confidence interval shaded) of the effect of number of dead standing agave stalks (*dens_dry*) within 30 m of the focal agave on per‐bat visitation rate at focal agaves.

Two local‐scale variables were also identified in the model set. The number of agaves with open flowers within 30 m of the focal agave was a significant predictor in seven models (Table S5), and the number of dead standing agave stalks within 30 m of the focal agave occurred in seven of the models and was significant in four. Visitation rate was negatively related to local abundance of agaves with open flowers (model‐averaged estimate: *β* = −0.628 ± 0.426 SE, Table S6, Figure 7) but was positively related to local abundance of agaves with dead standing agave stalks (model‐averaged estimate: *β* = 0.400 ± 0.297 SE, Table S6, Figure 8).

**FIGURE 9:**
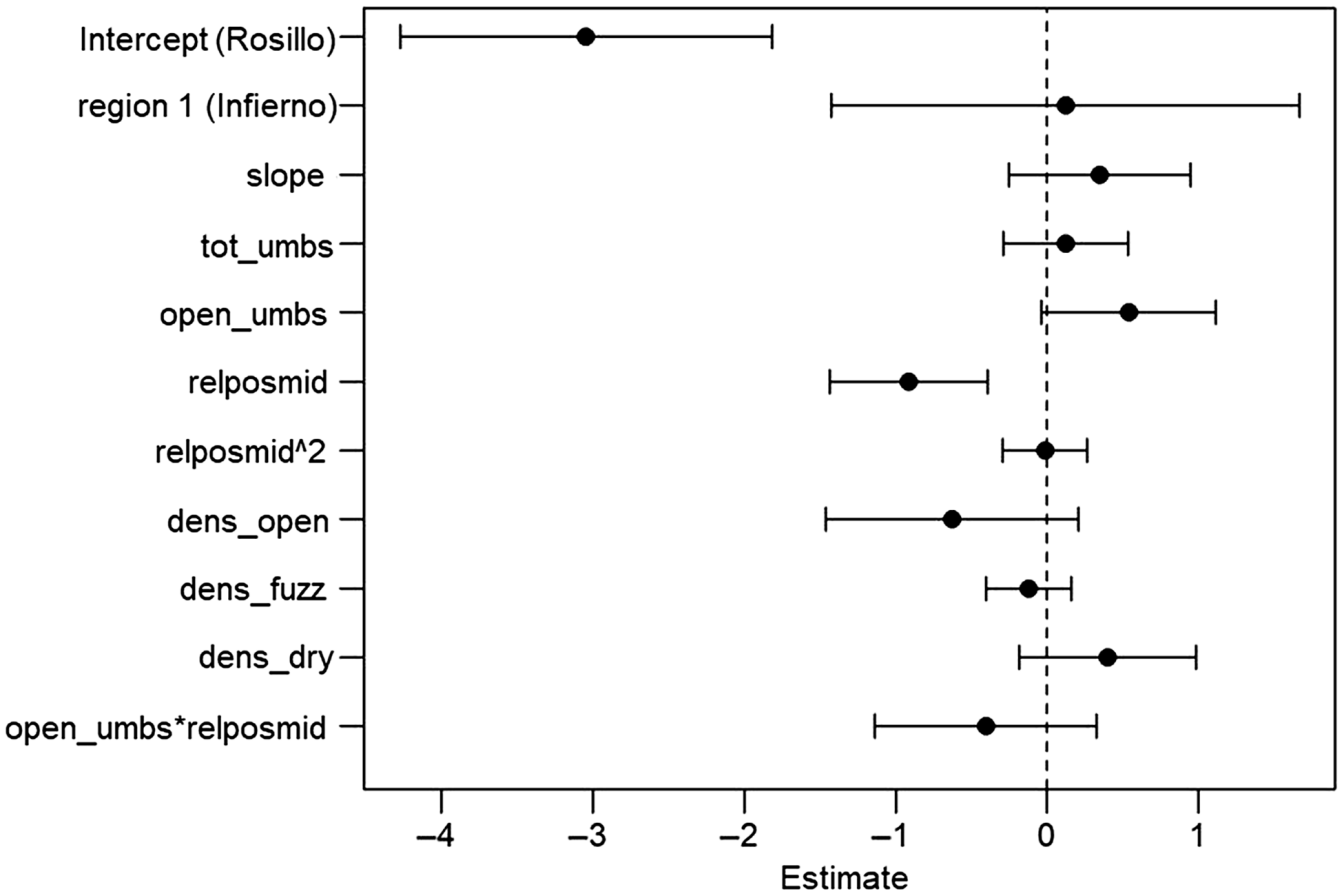
Standardized model‐averaged parameter estimates with their 95% confidence intervals. For reference: *tot_umbs* is the total number of umbels on the focal agave; *open_umbs* is the number of umbels with open flowers on the focal agave; *relposmid* is the relative vertical position of umbels with open flowers on the focal agave; *dens_open* is the number of agaves with open flowers within 30 m of the focal agave; *dens_fuzz* is the number of agaves with senescent flowers that no longer have available nectar within 30 m of the focal agave; and *dens_dry* is the number of dead standing agave stalks within 30 m of the focal agave.

The relative vertical position of umbels with open flowers was the strongest predictor of per‐bat visitation rate, as the model‐averaged confidence interval of no other predictor excluded zero (Figure 9). Other predictor variables (number of umbels with open flowers on the focal agave; density of open and dead standing agaves surrounding the focal agave; and the interaction between the number of umbels with open flowers and relative position on the stalk) were significant in some or all of the models, but none explained significant variation in visitation rate in the averaged model (no 95% confidence interval excluded zero). We found no significant relationships between per‐bat visitation rate and the remaining agave‐scale variables (total umbels on an agave and the quadratic term for relative position of umbels with open flowers or slope), local‐scale variable (number of agaves with senescent flowers), or region (Tables S5 and S6).

## 4 DISCUSSION

We recorded over seven times as many total bat visits (23,078) at the 28 agaves in the Infierno region as at the 34 agaves in the Rosillo region (3050). This is consistent with colony numbers at each roost, with Infierno Cave having an estimated 1000 bats based on emergence counts conducted in the mid‐2010s (E. Gómez‐Ruiz pers. comm) and Rosillo Cave having much lower numbers (U.S. Fish and Wildlife Service, 1988). While we did not assume constant colony sizes for the duration of the study, we did assume that Infierno Cave had consistently higher colony sizes than Rosillo Cave. Also, the average per‐bat visitation rate was 7 times higher at Infierno agaves (18.6 visits per bat per hour) than at Rosillo agaves (2.6 visits per bat per hour). However, the top‐ranked model did not include region as a covariate (Table 1). This may have been due to the use of a random effect to account for zero‐inflation or may indicate that the region does not explain additional variance in visitation rate beyond that which is explained by other covariates.

Our study identified several relationships between agave‐scale and local‐scale characteristics and per‐bat visitation rates for flowering agaves. Given the absence of rigorous hypothesis testing through manipulative experiments, these observational trends point to potentially useful management strategies that can be implemented and monitored as part of an adaptive management approach to aid in conservation efforts.

We found that the per‐bat visitation rate was positively associated with the number of umbels with open flowers, consistent with previous studies of flowering agaves (Ober & Steidl, 2004) and cacti (Arias‐Coyotl et al., 2006). While agave species identity was not considered in focal plant selection or as a factor in data analysis due to the inability to definitively distinguish species by visual inspection, these results suggest that agave species with higher numbers of umbels with simultaneously blooming flowers could enhance the attractiveness of foraging bats and could be targeted for planting in conservation programs aimed at increasing foraging resources for *L. nivalis*.

Our work shows the importance of considering flowering phenology when designing management to augment foraging resources for *L. nivalis*. As agave flowers open sequentially up the stalk, only a few umbels have open flowers with available nectar at a time. We found that visitation rate to be strongly positively associated with the relative position of umbels with open flowers along the stalk, with higher visitation rates at plants with lower‐positioned flowering umbels. This may indicate that early‐stage flowering plants were preferred over later‐stage plants. This relationship is unlikely to be due to the timing of agave flowering and the bats' migration through the area because at any given time throughout the study season there were both early and late phenological stage flowering plants present and monitored. Early‐stage flowering plants may be preferred over later‐stage plants because flowers on early‐stage plants may contain more nectar or nectar with a higher sugar concentration. Future studies could investigate nectar production, sugar concentration, and their phenological variation in the agave species found in the study area.

Furthermore, the interaction between the number of umbels with open flowers and their relative position on the stalk was also significant in explaining the per‐bat visitation rate. The number of umbels with open flowers strongly influenced the per‐bat visitation rate when they occurred at the bottom of the stalk. Conversely, the number of umbels with open flowers had little effect on the per‐bat visitation rate when they occurred at the top of the stalk, possibly due to less nectar availability or lower sugar concentration in higher flowers. Future studies could further investigate what makes lower‐positioned flowers more attractive to foraging bats.

Thus, both the available open flowers on a plant and the relative timing of flowering on individual plants appear to influence bat visitation. Augmenting the availability of flowering agaves and monitoring responses in bat visitation may therefore be a fruitful adaptive management strategy. Some of the agave species with the greatest abundance of open flowers at one time (e.g., *A. salmiana* in the Rosillo region and *A. montana* in the Infierno region) flower earlier in the season than other species, possibly completing flowering before the majority of *L. nivalis* arrives in the area (K. Lear, personal observation). Therefore, we stress the importance of considering the characteristics of both the floral architecture and phenology of agave species when formulating management recommendations for agave augmentation programs. While the earlier‐blooming species may not currently be a significant food source for migrating *L. nivalis* in our study area, such species may become increasingly important as the climate changes and the timing of the bats' migration potentially shifts earlier (Gómez‐Ruiz & Lacher, 2017). In order to increase the availability of agaves with lower‐positioned flowering umbels (i.e., in earlier phenological stages) at any point throughout the period when *L. nivalis* is present in northeast Mexico, multiple native agave species with different flowering times could be planted. Planting multiple agave species to span the full range of flowering times could therefore extend the period during which nectar is available to bats, helping to mitigate any potential temporal mismatches between bat migration and flower availability and creating more resilient plant‐pollinator food chains (Buckley & Nabhan, 2016; Gómez‐Ruiz & Lacher, 2019).

New approaches, such as eDNA (environmental DNA) analysis to detect bat DNA from agave flowers, are being investigated as survey methods for *L. nivalis* and other nectarivorous bats (Walker et al., 2022). Our finding that bat visitation was more strongly related to lower flower position when umbels with open flowers were abundant can also be used to design optimal eDNA field sampling protocols, for example, targeting sample collection from the lowest umbels with open flowers on a stalk.

Contrary to other studies of nectarivorous bats foraging at agaves (e.g., Ober & Steidl, 2004), the visitation rate at a focal agave was negatively related to the local density of agaves with open flowers. Areas with more flowering agaves provide more foraging resources than low‐density areas. Therefore, visits may be distributed over more plants, and the reduced visitation rate at any one focal agave may not be reflective of abundant food availability overall. Indeed, the average local density of agaves with open flowers in the Rosillo region was 1.5 times higher than in the Infierno region, while the per‐bat visitation rate was 7 times lower. On some nights, we observed the bats feeding on nearby agaves when not at the focal agave, suggesting that they visit multiple neighboring agaves. These observations are consistent with studies of *L. yerbabuenae* (e.g., Horner et al., 1998; Howell, 1979) and suggest that planting agaves in clusters may best match the bats' foraging behavior as well as reduce competition for food resources.

While the visitation rate at individual agaves may not be higher in higher‐density areas, Ober et al. (2005) observed higher densities of flowering agaves within the home ranges of *L. yerbabuenae* than elsewhere on the landscape in southeastern Arizona, suggesting that bats selected foraging areas with high resource availability. England (2012) also found that *L. nivalis* in Big Bend National Park in Texas centered their foraging activity in areas with high concentrations of nectar resources (*A. Havardiana*). Furthermore, Ober et al. (2005) found that forage resource density influenced the nightly activity budgets of *L. yerbabuenae*, with 120% more time spent foraging on average and 66% less time spent night‐roosting in a year when flowering agave density was lower than a year with more plentiful food resources. Reductions in densities of simultaneously flowering agaves could therefore increase the energy demands of foraging bats and potentially reduce their survival or fitness. Planting agaves in clusters may be most beneficial for reducing energy expenditures for bats while they forage.

We also found higher bat visitation rates at sites with higher densities of dead standing agave stalks, a signature of abundant flowering in previous years. During fieldwork, we observed visits to the same focal agaves over multiple consecutive nights and to some of the same sites over the 2 years of monitoring, suggesting that food resource history may drive foraging site fidelity nightly and perhaps yearly. Ober et al. (2005) observed nightly foraging fidelity in *L. yerbabuenae* and preferential selection of foraging areas with evidence of high food abundance in previous years (density of dead standing agave stalks). While data on the mechanism(s) driving foraging site fidelity in *Leptonycteris* are limited, site fidelity may arise from spatial memory of previous foraging areas and/or contemporary cues (e.g., visual or olfactory) provided by dead stalks. The former suggests the importance of targeting agave augmentation efforts toward sites where bats have been observed foraging previously, and the latter suggests the importance of keeping some high‐density areas of dead standing agave stalks on the landscape.

The spatial patterning of bat foraging also has strategic implications for the creation of multiple agave augmentation sites within a larger area. Previous studies found that *L. yerbabuenae* shifted foraging areas several hundred meters from night to night (Horner et al., 1998) and switched foraging areas when local flowers stopped producing nectar (Ober et al., 2005). These findings suggest that creating agave augmentation sites in areas surrounding known foraging locations may be an advantageous strategy to capitalize on bats' foraging behavior.

Several key uncertainties still exist regarding the foraging behavior and foraging requirements of *L. nivalis*. In our study, the relative vertical position of umbels with open flowers was the strongest predictor of the per‐bat visitation rate. Several other predictor variables showed mixed evidence for their relationship to bat visitation rate; thus, their effects on visitation rate remain uncertain. Targeting these variables in future studies could help reduce this uncertainty and provide more informed guidance for enhancing foraging resources for *L. nivalis*.

Another important uncertainty in conservation efforts aimed at augmenting foraging resources is the relative contribution of additional flowering agaves on the landscape to bat survival and population size. Key areas of future research to address this include energetics studies of *L. nivalis*, nectar production and concentration studies of agaves in northeast Mexico, agave population ecology modeling, and tracking of nightly foraging movements. Such data could inform agent‐based (or individual‐based) modeling of bat population dynamics that simulates resource availability and bat foraging behaviors. Monitoring to determine population trends will also be critical to reducing the key uncertainty about how management actions may affect bat survival and population status.

Our study provided insights into the relationship between bat visitation rates and agave‐ and local‐scale characteristics. These insights highlight important considerations for management actions that will ultimately shape ongoing conservation efforts for the species. Because our study did not rigorously test specific hypotheses through manipulative experiments, there may be alternative explanations for the patterns we observed, and thus the patterns can be used in an adaptive management framework where we can learn from the employment of potentially useful management strategies. In addition, we have not addressed the local social‐ecological contexts of target conservation areas, including local people's livelihood uses of agaves and their management practices, into which conservation efforts would be integrated. Conservation success is likely achievable only when bat conservation actions are designed to support both bat populations and local livelihoods. A holistic approach would consider local people as partners in conservation decision‐making. Results from this study can be incorporated into collaborative decision‐making processes that simultaneously consider conservation and livelihood needs.

## AUTHOR CONTRIBUTIONS

**Ana Castañeda Aguilera:** Investigation (equal); methodology (supporting). **Cuauhtemoc Ibarra Sanchez:** Investigation (equal); methodology (supporting). **Clinton T. Moore:** Conceptualization (equal); formal analysis (equal); methodology (equal); validation (equal); visualization (supporting); writing – original draft (supporting); writing – review and editing (equal). **Elizabeth G. King:** Conceptualization (equal); funding acquisition (supporting); methodology (equal); resources (equal); supervision (supporting); writing – original draft (supporting); writing – review and editing (equal). **Emma Gómez‐Ruiz:** Conceptualization (equal); methodology (equal); writing – review and editing (equal). **Jose Juan Flores Maldonado:** Conceptualization (equal); methodology (supporting); supervision (equal); writing – review and editing (supporting). **Jeff Hepinstall‐Cymerman:** Conceptualization (equal); funding acquisition (supporting); methodology (equal); resources (equal); supervision (supporting); writing – original draft (supporting); writing – review and editing (equal). **Kristen M. Lear:** Conceptualization (lead); data curation (lead); formal analysis (lead); funding acquisition (lead); investigation (lead); methodology (lead); project administration (lead); resources (equal); software (equal); supervision (equal); validation (lead); visualization (lead); writing – original draft (lead); writing – review and editing (equal). **Thomas J. Prebyl:** Formal analysis (supporting); methodology (equal); writing – review and editing (supporting).

## CONFLICT OF INTEREST STATEMENT

The authors have no conflicts of interest to declare.

### OPEN RESEARCH BADGES

This article has earned an Open Data badge for making publicly available the digitally‐shareable data necessary to reproduce the reported results. The data is available at https://knb.ecoinformatics.org/view/doi%3A10.5063%2FF1QJ7FST and https://doi.org/10.5063/F1QJ7FST.

## Supporting information


Data S1

## Data Availability

The data that support the findings of this study are openly available in Knowledge Network for Biocomplexity at https://doi.org/10.5063/F1QJ7FST (Lear, 2023).
