# Supplementary material for: Agave distribution and floral display influence foraging rates of an endangered pollinating bat and implications for conservation
Source: Ecol Evol. 2024 Mar 15;14(3):e11125. doi: 10.1002/ece3.11125 (PMC10941551; doi:10.1002/ece3.11125)
Supplement: Supplementary file 1 — Data S1 [file ECE3-14-e11125-s001.docx]

**Appendix**

| **Supporting Information**  **“Agave distribution and floral display influence foraging rates of an endangered pollinating bat and implications for conservation”**  **Lear et al.**  **Table S1.** Paniculate agave species that we found in our study sites and surrounding communities in each study region, with their scientific names (Gentry, 1982) and locally-recognized names.   \|  \| **Agave species (scientific name)** \| **Local names** \| \| --- \| --- \| --- \| \| **Infierno** \| *Agave americana* \| Maguey del castilla cenizo; Castilla cenizo; Maguey blanco de Castilla \| \| *Agave asperrima* \| Charchín; Cenizo del monte; Cenizo del campo \| \| *Agave salmiana* \| Maguey del castilla verde; Castilla verde \| \| *Agave gentryi* \| Maguey verde; Maguey verde del monte; Maguey verde del campo \| \| *Agave montana* \|  \| \| *Agave americana* \| Maguey cenizo; Cenizo forrajero; Maguey bronco \| \| **Rosillo** \| *Agave asperrima* \| Cenizo del monte \| \| *Agave salmiana* \| Maguey verde; Maguey manso; Cimarrón \| |
| --- | --- | --- | --- | --- | --- | --- | --- | --- | --- | --- | --- | --- | --- | --- | --- | --- | --- | --- | --- | --- | --- |

**Table S2.** Description and rationale for inclusion of predictor variables. The maximum number of bats seen at any focal agave on a night (*num_bats*) is also included here, since this value was used in the models to calculate the per-bat visitation rate. *Indicates variables that were dropped from the full analysis (because of collinearity issues (*dens_stlk* and *elev*), model convergence issues (*dens_young*), or measurement error (*height*)).

| **Spatial scale of the variable** | **Predictor  variable** | **Definition** | **Rationale for inclusion** |
| --- | --- | --- | --- |
| **Focal agave** | *tot_umbs* | Total number of umbels on the stalk. An umbel is a group of flowers at the end of a lateral branch. | Higher numbers of total umbels may indicate denser floral displays and be more attractive to foraging bats. This variable is also able to be manipulated by management. |
|  | *open_umbs* | Number of umbels containing open flowers with available nectar (i.e. flowers in the pre-dehiscent, dehiscent, or post-dehiscent stages) | Higher numbers of umbels with open flowers are expected to increase visitation rate, as found by Ober and Steidl (2004) and Kuban (1989) for *Leptonycteris yerbabuenae*. |
|  | *relposmid* | Relative vertical position along the stalk of umbels containing open flowers: The portion of the stalk with branches and umbels was divided into a vertical scale from 0 to 1, with 0 being the bottom. *relposmid* was measured based on the position of the vertical centerpoint of the flowering umbels | The position of umbels with open flowers on the stalk was shown to significantly affect visitation rates of *L. yerbabeunae* (Ober and Steidl 2004). |
|  | *height** | Height of the flowering stalk | Taller stalks may indicate denser floral displays and be more attractive to foraging bats. |
|  | *elev** | Elevation, as measured at each focal agave | Elevation may account for unmeasured variables (e.g. soil moisture) that could influence agave growth and flowering. In addition, sites can be selected for conservation activities based on this attribute. |
|  | *slope* | Slope, as estimated at each focal agave | Slope may account for unmeasured variables (e.g. soil moisture) that could influence agave growth and flowering. In addition, sites can be selected for conservation activities based on this attribute. |
| **Local scale (30 m radius around the focal agave)** | *dens_young** | Number of agave stalks with only young (unopened) flowers within 30 m of the focal agave | Higher densities of agave stalks may provide a visual cue to foraging bats as they search for food. |
|  | *dens_open* | Number of agave stalks with any umbels with open flowers within 30 m of the focal agave | Higher densities of flowering conspecifics may attract higher numbers of foraging bats. This variable may be manipulated by land management. |
|  | *dens_fuzz* | Number of agave stalks within 30 m of the focal agave with senescent flowers that no longer have available nectar | *L. yerbabuenae* exhibit foraging site fidelity across nights and likely across years. The density of agaves with senescent flowers provides evidence of food abundance. |
|  | *Dens_dry* | Number of dead (dry) standing agave stalks that had flowered in previous years within 30 m of the focal agave | *L. yerbabuenae* exhibit foraging site fidelity across nights and likely across years. The density of dead standing stalks provides evidence of food abundance in previous years and bats may preferentially select these areas when foraging (Ober et al. 2005). |
|  | *dens_stlk** | Number of any type of agave stalk within 30 m of the focal agave | Higher densities of agave stalks may provide a visual cue to foraging bats as they search for food. This variable may be manipulated by land management. |
|  | *reg* | Region (Infierno or Rosillo) | The two main roosting caves for *L. nivalis* in northeast Mexico are located in two regions. |
| **Other** | *num_bats* | The maximum number of bats seen (either visiting the focal agave or doing a "fly by") at any of the focal agaves monitored in a single night. | Incorporating the number of bats observed allows us to obtain a per-bat visitation measure, which is directly applicable to management efforts. |

**Table S3.** Descriptive statistics of variables used in analyses (and their modeling roles in parentheses), calculated from the complete data set for each region. *Indicates variables that were dropped from the full analysis because of collinearity or model convergence issues. Also provided are summary statistics of the total visits per hour (not on a per-bat basis) for each region, which was not included in modeling. Note that all fixed effects were scaled (z-score transformed) in the models so that the regression coefficients were comparable in magnitude and their effects were biologically comparable (Schielzeth 2010).

| **Region** | **Variable** | **Min** | **Max** | **Mean** | **SD** |
| --- | --- | --- | --- | --- | --- |
| **Infierno** | *visits per hour* | 0 | 571.6 | 74.7 | 114.7 |
|  | *visits per bat per hour* (response variable) | 0 | 119.3 | 18.6 | 24.5 |
|  | *tot_umbs* (predictor variable) | 10 | 35 | 19.3 | 6.5 |
|  | *open_umbs* (predictor variable) | 1 | 22 | 9.4 | 4.7 |
|  | *relposmid* (predictor variable) | 0.1 | 0.9 | 0.5 | 0.3 |
|  | *dens_stlk** (predictor variable) | 1 | 26 | 7.6 | 5.8 |
|  | *dens_young** (predictor variable) | 0 | 17 | 1.8 | 3.5 |
|  | *dens_open* (predictor variable) | 1 | 9 | 2.7 | 2.1 |
|  | *dens_fuzz* (predictor variable) | 0 | 3 | 0.8 | 1.1 |
|  | *dens_dry* (predictor variable) | 0 | 9 | 2.3 | 2.3 |
|  | *elev** (predictor variable) | 1859 | 2668 | 2063 | 260 |
|  | *slope* (predictor variable) | 0 | 60.0 | 26.6 | 17.5 |
|  | *num_bats* (used in the offset term) | 0 | 6 | 3.1 | 2.0 |
| **Rosillo** | *visits per hour* | 0 | 128.9 | 7.4 | 22.3 |
|  | *visits per bat per hour* (response variable) | 0 | 32.2 | 2.6 | 6.5 |
|  | *tot_umbs* (predictor variable) | 11 | 25 | 16.4 | 4.3 |
|  | *open_umbs* (predictor variable) | 2 | 15 | 6.1 | 2.9 |
|  | *relposmid* (predictor variable) | 0.1 | 0.9 | 0.6 | 0.3 |
|  | *dens_stlk** (predictor variable) | 1 | 34 | 9.1 | 7.7 |
|  | *dens_young** (predictor variable) | 0 | 9 | 1.2 | 2.4 |
|  | *dens_open* (predictor variable) | 1 | 13 | 4.0 | 3.2 |
|  | *dens_fuzz* (predictor variable) | 0 | 27 | 2.1 | 5.3 |
|  | *dens_dry* (predictor variable) | 0 | 10 | 1.7 | 2.8 |
|  | *elev** (predictor variable) | 1053 | 1907 | 1419 | 330 |
|  | *slope* (predictor variable) | 0 | 0.00 | 0.0 | 0.0 |
|  | *num_bats* (used in the offset term) | 0 | 4 | 0.8 | 1.3 |
| **Combined** | *visits per hour* | 0 | 571.6 | 37.0 | 84.4 |
|  | *visits per bat per hour* (response variable) | 0 | 119.3 | 9.6 | 18.7 |
|  | *tot_umbs* (predictor variable) | 10 | 35 | 17.7 | 5.6 |
|  | *open_umbs* (predictor variable) | 1 | 22 | 7.6 | 4.1 |
|  | *relposmid* (predictor variable) | 0.1 | 0.9 | 0.6 | 0.3 |
|  | *dens_stlk** (predictor variable) | 1 | 34 | 8.5 | 6.9 |
|  | *dens_young** (predictor variable) | 0 | 17 | 1.5 | 2.9 |
|  | *dens_open* (predictor variable) | 1 | 13 | 3.5 | 2.9 |
|  | *dens_fuzz* (predictor variable) | 0 | 27 | 1.5 | 4.1 |
|  | *dens_dry* (predictor variable) | 0 | 10 | 1.9 | 2.6 |
|  | *elev** (predictor variable) | 1053 | 2668 | 1702 | 439 |
|  | *slope* (predictor variable) | 0 | 60.0 | 11.7 | 17.6 |
|  | *num_bats* (used in the offset term) | 0 | 6 | 1.8 | 1.9 |

**Table S4.** Site-level descriptive statistics of the response variable used in analysis (visits per bat per hour) and predictor variables. *Indicates variables that were dropped from the full analysis because of collinearity issues. Also provided are site-level summary statistics of the total visits per hour (not on a per-bat basis). This variable was not included in analysis.

| **Site** | **Survey dates** | **Variable** | **Min** | **Max** | **Mean** | **SD** |
| --- | --- | --- | --- | --- | --- | --- |
| **Region: Infierno** | | | | | | |
| "Can" | 6/14/17; 6/14/18; 6/15/18 | *visits per hour* | 0 | 13.5 | 4.7 | 5.9 |
|  |  | *visits per bat per hour* (response variable) | 0 | 13.5 | 4.7 | 5.9 |
|  |  | *tot_umbs* (predictor variable) | 11.0 | 29.0 | 24.7 | 7.0 |
|  |  | *open_umbs* (predictor variable) | 7.0 | 11.0 | 8.3 | 2.1 |
|  |  | *relposmid* (predictor variable) | 0.5 | 1.0 | 0.8 | 0.2 |
|  |  | *dens_stlk** (predictor variable) | 1.0 | 5.0 | 3.8 | 1.5 |
|  |  | *dens_young* (predictor variable) | 0 | 0 | 0 | 0 |
|  |  | *dens_open* (predictor variable) | 1.0 | 2.0 | 1.2 | 0.4 |
|  |  | *dens_fuzz* (predictor variable) | 0 | 1.0 | 0.3 | 0.5 |
|  |  | *dens_dry* (predictor variable) | 0 | 3.0 | 2.3 | 1.2 |
|  |  | *elev** (predictor variable) | 2412 | 2668 | 2553 | 116 |
|  |  | *slope* (predictor variable) | 30.0 | 60.0 | 43.3 | 13.7 |
|  |  | *num_bats* (used in the offset term) | 0 | 1.0 | 0.7 | 0.5 |
| "Cas" | 6/7/17; 6/8/17 | *visits per hour* | 0 | 0 | 0 | 0 |
|  |  | *visits per bat per hour* (response variable) | 0 | 0 | 0 | 0 |
|  |  | *tot_umbs* (predictor variable) | 18.0 | 21.0 | 19.5 | 1.7 |
|  |  | *open_umbs* (predictor variable) | 10.0 | 10.0 | 10.0 | 0 |
|  |  | *relposmid* (predictor variable) | 0.3 | 0.9 | 0.6 | 0.3 |
|  |  | *dens_stlk** (predictor variable) | 3.0 | 4.0 | 3.5 | 0.6 |
|  |  | *dens_young* (predictor variable) | 0 | 0 | 0 | 0 |
|  |  | *dens_open* (predictor variable) | 2.0 | 2.0 | 2.0 | 0 |
|  |  | *dens_fuzz* (predictor variable) | 0 | 0 | 0 | 0 |
|  |  | *dens_dry* (predictor variable) | 1.0 | 2.0 | 1.5 | 0.6 |
|  |  | *elev** (predictor variable) | 2555 | 2642 | 2599 | 50 |
|  |  | *slope* (predictor variable) | 40.0 | 45.0 | 42.5 | 2.9 |
|  |  | *num_bats* (used in the offset term) | 0 | 0 | 0 | 0 |
| "Isa" | 7/30/17; 7/31/17; 7/12/18; 7/13/18; 7/25/18; 7/26/18; 7/27/18; 7/28/18 | *visits per hour* | 0.5 | 571.6 | 151.3 | 152.0 |
|  |  | *visits per bat per hour* (response variable) | 0.1 | 95.3 | 26.5 | 25.2 |
|  |  | *tot_umbs* (predictor variable) | 11.0 | 27.0 | 21.1 | 5.5 |
|  |  | *open_umbs* (predictor variable) | 7.0 | 22.0 | 12.2 | 4.1 |
|  |  | *relposmid* (predictor variable) | 0.3 | 0.9 | 0.5 | 0.2 |
|  |  | *dens_stlk** (predictor variable) | 3.0 | 26.0 | 11.9 | 7.0 |
|  |  | *dens_young* (predictor variable) | 0.0 | 17.0 | 3.7 | 5.0 |
|  |  | *dens_open* (predictor variable) | 1.0 | 9.0 | 4.2 | 2.7 |
|  |  | *dens_fuzz* (predictor variable) | 0 | 1.0 | 0.3 | 0.5 |
|  |  | *dens_dry* (predictor variable) | 0 | 9.0 | 3.6 | 2.9 |
|  |  | *elev** (predictor variable) | 1859 | 1933 | 1885 | 24 |
|  |  | *slope* (predictor variable) | 0 | 45.0 | 15.0 | 14.5 |
|  |  | *num_bats* (used in the offset term) | 4.0 | 6.0 | 5.4 | 0.7 |
| "Pen" | 6/5/17; 6/6/17; 6/28/17; 6/29/17; 6/30/17; 6/22/18; 6/23/18 | *visits per hour* | 0 | 121.6 | 37.5 | 39.0 |
|  |  | *visits per bat per hour* (response variable) | 0 | 55.6 | 15.1 | 15.5 |
|  |  | *tot_umbs* (predictor variable) | 10.0 | 19.0 | 13.7 | 2.7 |
|  |  | *open_umbs* (predictor variable) | 1.0 | 8.0 | 5.5 | 1.6 |
|  |  | *relposmid* (predictor variable) | 0.3 | 1.0 | 0.5 | 0.3 |
|  |  | *dens_stlk** (predictor variable) | 2.0 | 11.0 | 6.6 | 3.2 |
|  |  | *dens_young* (predictor variable) | 0 | 4.0 | 1.3 | 1.4 |
|  |  | *dens_open* (predictor variable) | 1.0 | 4.0 | 2.2 | 1.0 |
|  |  | *dens_fuzz* (predictor variable) | 0 | 3.0 | 1.6 | 1.5 |
|  |  | *dens_dry* (predictor variable) | 0 | 4.0 | 1.5 | 1.5 |
|  |  | *elev** (predictor variable) | 1932 | 2028 | 1962 | 26 |
|  |  | *slope* (predictor variable) | 20.0 | 45.0 | 33.9 | 9.4 |
|  |  | *num_bats* (used in the offset term) | 2.0 | 3.0 | 2.5 | 0.5 |
| "Raf" | 5/26/18; 5/27/18 | *visits per hour* | 1.3 | 4.7 | 2.5 | 1.6 |
|  |  | *visits per bat per hour* (response variable) | 1.3 | 4.7 | 2.5 | 1.6 |
|  |  | *tot_umbs* (predictor variable) | 21.0 | 35.0 | 28.0 | 8.1 |
|  |  | *open_umbs* (predictor variable) | 11.0 | 22.0 | 16.5 | 6.4 |
|  |  | *relposmid* (predictor variable) | 0.7 | 0.8 | 0.8 | 0.1 |
|  |  | *dens_stlk** (predictor variable) | 2.0 | 3.0 | 2.5 | 0.6 |
|  |  | *dens_young* (predictor variable) | 0 | 0 | 0 | 0 |
|  |  | *dens_open* (predictor variable) | 2.0 | 2.0 | 2.0 | 0 |
|  |  | *dens_fuzz* (predictor variable) | 0 | 1.0 | 0.5 | 0.6 |
|  |  | *dens_dry* (predictor variable) | 0 | 0 | 0 | 0 |
|  |  | *elev** (predictor variable) | 2217 | 2222 | 2219 | 3 |
|  |  | *slope* (predictor variable) | 0 | 0 | 0 | 0 |
|  |  | *num_bats* (used in the offset term) | 1.0 | 1.0 | 1.0 | 0 |
| "Teh" | 4/28/18 | *visits per hour* | 90.4 | 238.6 | 164.5 | 104.8 |
|  |  | *visits per bat per hour* (response variable) | 45.2 | 119.3 | 82.3 | 52.4 |
|  |  | *tot_umbs* (predictor variable) | 20.0 | 20.0 | 20.0 | 0 |
|  |  | *open_umbs* (predictor variable) | 4.0 | 8.0 | 6.0 | 2.8 |
|  |  | *relposmid* (predictor variable) | 0.1 | 0.3 | 0.2 | 0.1 |
|  |  | *dens_stlk** (predictor variable) | 4.0 | 4.0 | 4.0 | 0 |
|  |  | *dens_young* (predictor variable) | 0 | 0 | 0 | 0 |
|  |  | *dens_open* (predictor variable) | 1.0 | 1.0 | 1.0 | 0 |
|  |  | *dens_fuzz* (predictor variable) | 0 | 0 | 0 | 0 |
|  |  | *dens_dry* (predictor variable) | 2.0 | 3.0 | 2.5 | 0.7 |
|  |  | *elev** (predictor variable) | 1935 | 1950 | 1942 | 11 |
|  |  | *slope* (predictor variable) | 45.0 | 45.0 | 45.0 | 0 |
|  |  | *num_bats* (used in the offset term) | 2.0 | 2.0 | 2.0 | 0 |
| **Region: Rosillo** | | | | | | |
| "Bon" | 7/19/17; 7/20/17; 7/5/18; 7/7/18 | *visits per hour* | 0 | 128.9 | 40.5 | 40.6 |
|  |  | *visits per bat per hour* (response variable) | 0 | 32.2 | 13.0 | 10.6 |
|  |  | *tot_umbs* (predictor variable) | 12.0 | 18.0 | 15.0 | 2.0 |
|  |  | *open_umbs* (predictor variable) | 4.0 | 6.0 | 4.7 | 0.8 |
|  |  | *relposmid* (predictor variable) | 0.8 | 1.0 | 0.9 | 0.1 |
|  |  | *dens_stlk** (predictor variable) | 1.0 | 34.0 | 10.5 | 12.0 |
|  |  | *dens_young* (predictor variable) | 0 | 1.0 | 0.2 | 0.4 |
|  |  | *dens_open* (predictor variable) | 1.0 | 6.0 | 2.3 | 1.9 |
|  |  | *dens_fuzz* (predictor variable) | 0 | 27.0 | 6.8 | 10.1 |
|  |  | *dens_dry* (predictor variable) | 0 | 4.0 | 1.2 | 1.7 |
|  |  | *elev** (predictor variable) | 1416 | 1443 | 1425 | 9 |
|  |  | *slope* (predictor variable) | 0 | 0 | 0 | 0 |
|  |  | *num_bats* (used in the offset term) | 1.0 | 4.0 | 2.8 | 1.4 |
| "Cerc" | 6/7/18; 6/8/18; 6/9/18 | *visits per hour* | 0 | 0 | 0 | 0 |
|  |  | *visits per bat per hour* (response variable) | 0 | 0 | 0 | 0 |
|  |  | *tot_umbs* (predictor variable) | 12.0 | 15.0 | 13.0 | 1.5 |
|  |  | *open_umbs* (predictor variable) | 6.0 | 10.0 | 7.7 | 1.8 |
|  |  | *relposmid* (predictor variable) | 0.5 | 0.7 | 0.6 | 0.1 |
|  |  | *dens_stlk** (predictor variable) | 9.0 | 21.0 | 13.3 | 5.8 |
|  |  | *dens_young* (predictor variable) | 0 | 6.0 | 2.3 | 2.8 |
|  |  | *dens_open* (predictor variable) | 4.0 | 13.0 | 7.7 | 4.1 |
|  |  | *dens_fuzz* (predictor variable) | 0 | 0 | 0 | 0 |
|  |  | *dens_dry* (predictor variable) | 2.0 | 5.0 | 3.3 | 1.3 |
|  |  | *elev** (predictor variable) | 1193 | 1208 | 1201 | 7 |
|  |  | *slope* (predictor variable) | 0 | 0 | 0 | 0 |
|  |  | *num_bats* (used in the offset term) | 0 | 0 | 0 | 0 |
| "Dol" | 5/10/18; 5/11/18; 5/12/18; 5/18/18;  5/19/18; 6/2/18; 6/3/18 | *visits per hour* | 0 | 9.7 | 1.6 | 2.6 |
|  |  | *visits per bat per hour* (response variable) | 0 | 9.7 | 1.0 | 2.2 |
|  |  | *tot_umbs* (predictor variable) | 11.0 | 21.0 | 14.8 | 3.1 |
|  |  | *open_umbs* (predictor variable) | 2.0 | 8.0 | 5.3 | 1.7 |
|  |  | *relposmid* (predictor variable) | 0.1 | 0.6 | 0.3 | 0.1 |
|  |  | *dens_stlk** (predictor variable) | 2.0 | 13.0 | 6.3 | 3.8 |
|  |  | *dens_young* (predictor variable) | 0 | 9.0 | 2.6 | 3.2 |
|  |  | *dens_open* (predictor variable) | 1.0 | 6.0 | 3.3 | 1.6 |
|  |  | *dens_fuzz* (predictor variable) | 0 | 1.0 | 0.2 | 0.4 |
|  |  | *dens_dry* (predictor variable) | 0 | 2.0 | 0.2 | 0.6 |
|  |  | *elev** (predictor variable) | 1053 | 1135 | 1086 | 23 |
|  |  | *slope* (predictor variable) | 0 | 0 | 0 | 0 |
|  |  | *num_bats* (used in the offset term) | 0 | 3.0 | 1.0 | 1.1 |
| "Flet" | 7/5/17; 6/1/18 | *visits per hour* | 0 | 0 | 0 | 0 |
|  |  | *visits per bat per hour* (response variable) | 0 | 0 | 0 | 0 |
|  |  | *tot_umbs* (predictor variable) | 12.0 | 19.0 | 14.6 | 2.4 |
|  |  | *open_umbs* (predictor variable) | 4.0 | 6.0 | 4.6 | 0.8 |
|  |  | *relposmid* (predictor variable) | 0.2 | 0.7 | 0.5 | 0.2 |
|  |  | *dens_stlk** (predictor variable) | 7.0 | 31.0 | 15.6 | 8.7 |
|  |  | *dens_young* (predictor variable) | 0 | 4.0 | 0.9 | 1.6 |
|  |  | *dens_open* (predictor variable) | 3.0 | 11.0 | 6.4 | 3.0 |
|  |  | *dens_fuzz* (predictor variable) | 0 | 19.0 | 4.1 | 7.0 |
|  |  | *dens_dry* (predictor variable) | 0 | 10.0 | 4.1 | 4.9 |
|  |  | *elev** (predictor variable) | 1246 | 1298 | 1283 | 19 |
|  |  | *slope* (predictor variable) | 0 | 0 | 0 | 0 |
|  |  | *num_bats* (used in the offset term) | 0 | 0 | 0 | 0 |
| "GV" | 5/26/17; 5/28/17; 5/4/18; 5/5/18; 5/6/18 | *visits per hour* | 0 | 0 | 0 | 0 |
|  |  | *visits per bat per hour* (response variable) | 0 | 0 | 0 | 0 |
|  |  | *tot_umbs* (predictor variable) | 19.0 | 25.0 | 22.8 | 1.9 |
|  |  | *open_umbs* (predictor variable) | 2.0 | 15.0 | 8.1 | 4.8 |
|  |  | *relposmid* (predictor variable) | 0.7 | 1.0 | 0.8 | 0.1 |
|  |  | *dens_stlk** (predictor variable) | 1.0 | 17.0 | 8.4 | 6.3 |
|  |  | *dens_young* (predictor variable) | 0 | 0 | 0 | 0 |
|  |  | *dens_open* (predictor variable) | 1.0 | 11.0 | 3.6 | 3.7 |
|  |  | *dens_fuzz* (predictor variable) | 0 | 5.0 | 1.8 | 2.1 |
|  |  | *dens_dry* (predictor variable) | 0 | 9.0 | 2.6 | 3.6 |
|  |  | *elev** (predictor variable) | 1887 | 1907 | 1890 | 6 |
|  |  | *slope* (predictor variable) | 0 | 0 | 0 | 0 |
|  |  | *num_bats* (used in the offset term) | 0 | 0 | 0 | 0 |
| "Zac" | 6/23/17; 6/24/17 | *visits per hour* | 0 | 1.9 | 0.5 | 0.9 |
|  |  | *visits per bat per hour* (response variable) | 0 | 1.9 | 0.5 | 0.9 |
|  |  | *tot_umbs* (predictor variable) | 12.0 | 15.0 | 13.5 | 1.7 |
|  |  | *open_umbs* (predictor variable) | 5.0 | 6.0 | 5.5 | 0.6 |
|  |  | *relposmid* (predictor variable) | 0.2 | 0.9 | 0.5 | 0.4 |
|  |  | *dens_stlk** (predictor variable) | 2.0 | 3.0 | 2.5 | 0.6 |
|  |  | *dens_young* (predictor variable) | 0 | 0 | 0 | 0 |
|  |  | *dens_open* (predictor variable) | 2.0 | 3.0 | 2.5 | 0.6 |
|  |  | *dens_fuzz* (predictor variable) | 0 | 0 | 0 | 0 |
|  |  | *dens_dry* (predictor variable) | 0 | 0 | 0 | 0 |
|  |  | *elev** (predictor variable) | 1877 | 1880 | 1878 | 2 |
|  |  | *slope* (predictor variable) | 0 | 0 | 0 | 0 |
|  |  | *num_bats* (used in the offset term) | 0 | 1.0 | 0.5 | 0.6 |

**Table S5.** Model summary of standardized parameter estimates (β) and standard errors (SE) of the predictor variables explaining per-bat visitation rate from all models in the 95% confidence set (i.e. the top 13 models from Table 2.4). "NA" denotes parameters that are not present in the model. Significant parameter estimates for each model (p < 0.05) are shown in bold.

|  | **Intercept** | **reg** | **slope** | **tot_umbs** | **open_umbs** | **relposmid** | **dens_open** | **dens_fuzz** | **dens_dry** | **open_umbs*relposmid** | **relposmid^2^** |
| --- | --- | --- | --- | --- | --- | --- | --- | --- | --- | --- | --- |
| ***Model 1*** |  |  |  |  |  |  |  |  |  |  |  |
| β | **-3.018** | NA | 0.445 | 0.157 | **0.618** | **-1.000** | **-0.831** | -0.145 | **0.544** | **-0.625** | NA |
| ±SE | 0.441 | NA | 0.253 | 0.219 | 0.244 | 0.242 | 0.289 | 0.141 | 0.226 | 0.269 | NA |
| *z* value | -6.849 | NA | 1.755 | 0.718 | 2.530 | -4.139 | -2.872 | -1.027 | 2.412 | -2.327 | NA |
| p value | <0.001 | NA | 0.079 | 0.473 | 0.011 | <0.001 | 0.004 | 0.304 | 0.016 | 0.020 | NA |
| ***Model 2*** |  |  |  |  |  |  |  |  |  |  |  |
| β | **-2.750** | -0.498 | 0.532 | 0.179 | **0.665** | **-1.018** | **-0.876** | -0.141 | **0.551** | **-0.661** | NA |
| ±SE | 0.692 | 1.043 | 0.308 | 0.221 | 0.260 | 0.241 | 0.302 | 0.140 | 0.223 | 0.277 | NA |
| *z* value | -3.974 | -0.477 | 1.724 | 0.810 | 2.564 | -4.230 | -2.903 | -1.006 | 2.476 | -2.389 | NA |
| p value | <0.001 | 0.633 | 0.085 | 0.418 | 0.010 | <0.001 | 0.004 | 0.314 | 0.013 | 0.017 | NA |
| ***Model 3*** |  |  |  |  |  |  |  |  |  |  |  |
| β | **-2.984** | NA | 0.445 | 0.161 | **0.603** | **-1.001** | **-0.832** | -0.146 | **0.538** | **-0.619** | -0.030 |
| ±SE | 0.557 | NA | 0.253 | 0.222 | 0.289 | 0.242 | 0.289 | 0.141 | 0.233 | 0.275 | 0.297 |
| *z* value | -5.358 | NA | 1.756 | 0.726 | 2.089 | -4.145 | -2.877 | -1.034 | 2.305 | -2.250 | -0.100 |
| p value | <0.001 | NA | 0.079 | 0.468 | 0.037 | <0.001 | 0.004 | 0.301 | 0.021 | 0.024 | 0.921 |
| ***Model 4*** |  |  |  |  |  |  |  |  |  |  |  |
| β | **-3.094** | NA | 0.341 | 0.132 | **0.529** | -0.795 | **-0.627** | -0.197 | 0.373 | NA | NA |
| ±SE | 0.490 | NA | 0.266 | 0.230 | 0.250 | 0.227 | 0.296 | 0.143 | 0.223 | NA | NA |
| *z* value | -6.319 | NA | 1.286 | 0.573 | 2.111 | -3.509 | -2.121 | -1.377 | 1.668 | NA | NA |
| p value | <0.001 | NA | 0.199 | 0.566 | 0.035 | <0.001 | 0.034 | 0.169 | 0.095 | NA | NA |
| ***Model 5*** |  |  |  |  |  |  |  |  |  |  |  |
| β | **-3.692** | 1.564 | NA | NA | 0.259 | **-0.716** | NA | NA | NA | NA | NA |
| ±SE | 0.731 | 0.940 | NA | NA | 0.255 | 0.225 | NA | NA | NA | NA | NA |
| *z* value | -5.052 | 1.664 | NA | NA | 1.016 | -3.189 | NA | NA | NA | NA | NA |
| p value | <0.001 | 0.096 | NA | NA | 0.309 | 0.001 | NA | NA | NA | NA | NA |
| ***Model 6*** |  |  |  |  |  |  |  |  |  |  |  |
| β | **-2.887** | NA | NA | NA | 0.382 | **-0.734** | NA | NA | NA | NA | NA |
| ±SE | 0.516 | NA | NA | NA | 0.241 | 0.224 | NA | NA | NA | NA | NA |
| *z* value | -5.592 | NA | NA | NA | 1.585 | -3.278 | NA | NA | NA | NA | NA |
| p value | <0.001 | NA | NA | NA | 0.113 | 0.001 | NA | NA | NA | NA | NA |
| ***Model 7*** |  |  |  |  |  |  |  |  |  |  |  |
| β | **-2.896** | NA | 0.344 | 0.159 | 0.444 | **-0.816** | **-0.635** | -0.205 | 0.347 | NA | -0.173 |
| ±SE | 0.595 | NA | 0.265 | 0.232 | 0.289 | 0.228 | 0.292 | 0.142 | 0.227 | NA | 0.296 |
| *z* value | -4.866 | NA | 1.300 | 0.684 | 1.536 | -3.583 | -2.175 | -1.450 | 1.531 | NA | -0.584 |
| p value | <0.001 | NA | 0.194 | 0.494 | 0.125 | <0.001 | 0.030 | 0.147 | 0.126 | NA | 0.559 |
| ***Model 8*** |  |  |  |  |  |  |  |  |  |  |  |
| β | **-2.742** | -0.493 | 0.531 | 0.180 | **0.660** | **-1.018** | **-0.876** | -0.141 | **0.549** | **-0.659** | -0.010 |
| ±SE | 0.740 | 1.054 | 0.310 | 0.224 | 0.311 | 0.241 | 0.302 | 0.140 | 0.231 | 0.286 | 0.297 |
| *z* value | -3.707 | -0.468 | 1.713 | 0.805 | 2.121 | -4.230 | -2.902 | -1.005 | 2.379 | -2.305 | -0.033 |
| p value | <0.001 | 0.640 | 0.087 | 0.421 | 0.034 | <0.001 | 0.004 | 0.315 | 0.017 | 0.021 | 0.973 |
| ***Model 9*** |  |  |  |  |  |  |  |  |  |  |  |
| β | **-3.247** | 0.297 | 0.296 | 0.119 | 0.509 | **-0.791** | **-0.610** | -0.198 | 0.374 | NA | NA |
| ±SE | 0.785 | 1.157 | 0.319 | 0.236 | 0.264 | 0.229 | 0.306 | 0.144 | 0.225 | NA | NA |
| *z* value | -4.138 | 0.256 | 0.928 | 0.504 | 1.931 | -3.459 | -1.994 | -1.372 | 1.664 | NA | NA |
| p value | <0.001 | 0.798 | 0.354 | 0.614 | 0.054 | <0.001 | 0.046 | 0.170 | 0.096 | NA | NA |
| ***Model 10*** |  |  |  |  |  |  |  |  |  |  |  |
| β | **-3.611** | 1.535 | NA | NA | 0.249 | **-0.762** | NA | NA | NA | -0.201 | NA |
| ±SE | 0.705 | 0.901 | NA | NA | 0.261 | 0.234 | NA | NA | NA | 0.273 | NA |
| *z* value | -5.121 | 1.704 | NA | NA | 0.956 | -3.253 | NA | NA | NA | -0.737 | NA |
| p value | <0.001 | 0.088 | NA | NA | 0.339 | 0.001 | NA | NA | NA | 0.461 | NA |
| ***Model 11*** |  |  |  |  |  |  |  |  |  |  |  |
| β | **-3.554** | 1.601 | NA | NA | 0.208 | **-0.732** | NA | NA | NA | NA | -0.135 |
| ±SE | 0.794 | 0.951 | NA | NA | 0.280 | 0.226 | NA | NA | NA | NA | 0.299 |
| *z* value | -4.477 | 1.683 | NA | NA | 0.741 | -3.234 | NA | NA | NA | NA | -0.450 |
| p value | <0.001 | 0.092 | NA | NA | 0.459 | 0.001 | NA | NA | NA | NA | 0.653 |
| ***Model 12*** |  |  |  |  |  |  |  |  |  |  |  |
| β | **-2.831** | NA | NA | NA | 0.390 | **-0.781** | NA | NA | NA | -0.174 | NA |
| ±SE | 0.502 | NA | NA | NA | 0.246 | 0.237 | NA | NA | NA | 0.272 | NA |
| *z* value | -5.636 | NA | NA | NA | 1.585 | -3.291 | NA | NA | NA | -0.640 | NA |
| p value | <0.001 | NA | NA | NA | 0.113 | <0.001 | NA | NA | NA | 0.522 | NA |
| ***Model 13*** |  |  |  |  |  |  |  |  |  |  |  |
| β | **-2.768** | NA | NA | NA | 0.342 | **-0.749** | NA | NA | NA | NA | -0.105 |
| ±SE | 0.617 | NA | NA | NA | 0.266 | 0.227 | NA | NA | NA | NA | 0.295 |
| *z* value | -4.485 | NA | NA | NA | 1.286 | -3.296 | NA | NA | NA | NA | -0.356 |
| p value | <0.001 | NA | NA | NA | 0.198 | <0.001 | NA | NA | NA | NA | 0.722 |

**Table S6.** Standardized model-averaged parameter estimates using the 95% confidence set of models (i.e. the top 13 models). Both standard errors (SE) and 95% confidence intervals (95% CI) are given. Parameters whose 95% confidence intervals do not overlap zero are shown in bold. Unstandardized model-averaged parameter estimates are denoted with *.

|  | **Intercept** | **reg** | **slope** | **tot_umbs** | **open_umbs** | **relposmid** | **dens_open** | **dens_fuzz** | **dens_dry** | **open_umbs*relposmid** | **relposmid^2^** |
| --- | --- | --- | --- | --- | --- | --- | --- | --- | --- | --- | --- |
| β | **-3.044** | 0.121 | 0.345 | 0.124 | 0.539 | **-0.914** | -0.628 | -0.122 | 0.400 | -0.405 | -0.014 |
| SE | 0.626 | 0.788 | 0.306 | 0.210 | 0.294 | 0.266 | 0.426 | 0.143 | 0.297 | 0.373 | 0.143 |
| 95% CI | (-4.270,  -1.818) | (-1.423, 1.665) | (-0.254, 0.944) | (-0.288, 0.536) | (-0.037, 1.115) | (-1.436,  -0.392) | (-1.464, 0.208) | (-0.402, 0.158) | (-0.183, 0.983) | (-1.136, 0.326) | (-0.294, 0.266) |
| β * | -3.839 | 0.121 | 0.020 | 0.022 | 0.338 | -0.431 | -0.220 | -0.030 | 0.154 | -0.366 | -0.197 |


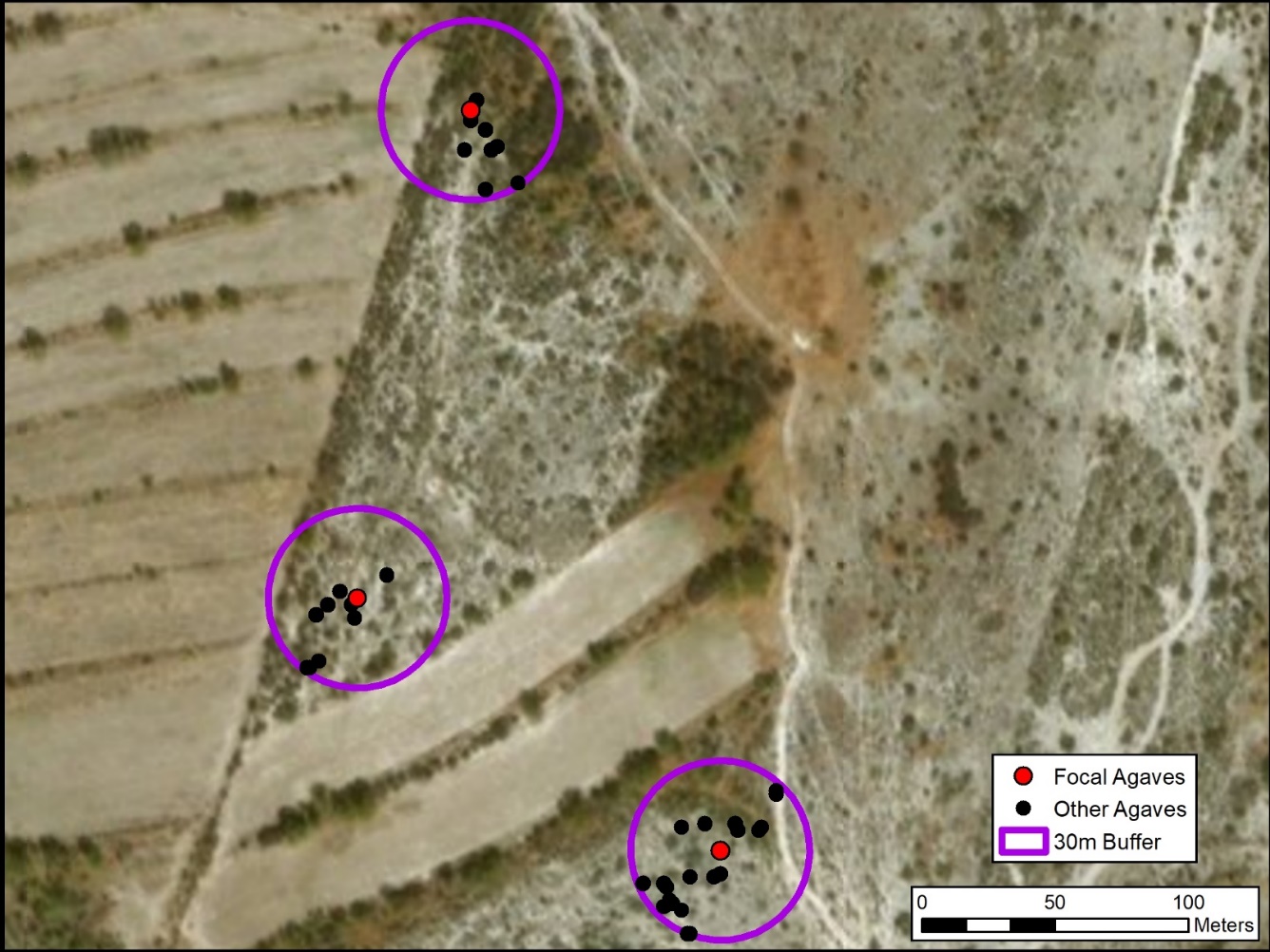


**Figure S1.** A representative cluster of three focal agaves (red dots) that were monitored concurrently within one site in the Rosillo region. All other agaves with any type of stalk within 30 m of each focal agave are shown as black dots. The main vegetation in this area was mesquite and creosote bush. Aerial imagery from Maxar Vivid Imagery (10/17/2016).


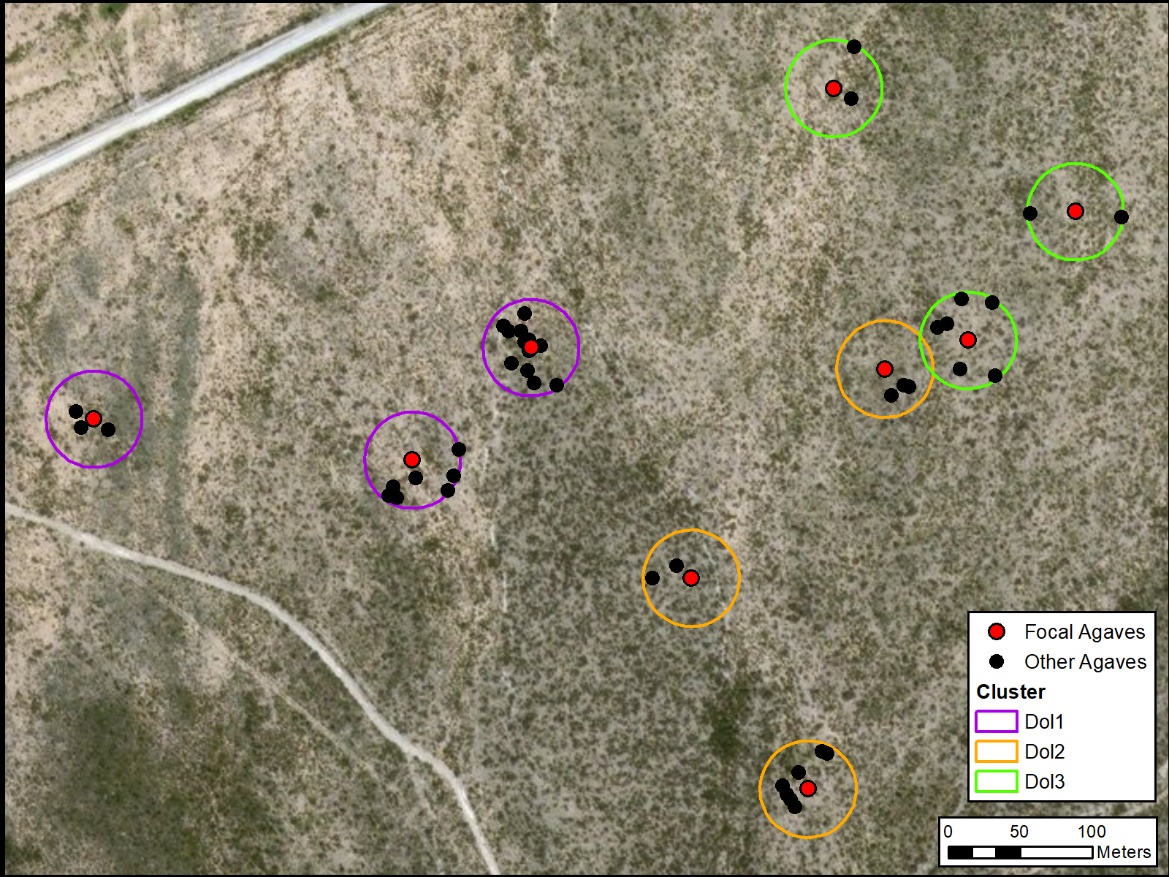


**Figure S2.** A representative site (“Dol”) in the Rosillo region with multiple clusters of focal agaves (red dots). Each cluster is shown in a different color. The focal agaves in each cluster were monitored simultaneously, and the clusters were monitored on different nights. All other agaves with any type of stalk within 30 m of each focal agave are shown as black dots. Aerial imagery from Maxar Vivid Imagery (10/17/2016).

R CODE USED FOR STATISTICAL MODELING

The following code was run through R to perform statistical modeling of bat visitation rates to flowering agaves. This R code and data files can be found on the Knowledge Network for Biocomplexity at <https://doi.org/10.5063/F1QJ7FST>.

Kristen Lear. (2023). Nectar bat foraging data at flowering agaves, Nuevo Leon and Coahuila, Mexico, May 26-July 31, 2017 and April 28-July 28, 2018 (Lear et al.). Knowledge Network for Biocomplexity. urn:uuid:acfee400-d241-4044-b68f-435324c8df05.

##############################Start of Code############################

######################################################################

###Load packages; import and configure data###

######################################################################

library(car)

library(MASS)

library(corrplot)

library(plyr)

library(dplyr)

library(reshape)

library(reshape2)

library(lme4)

library(lattice)

library(lmerTest)

library(sjPlot)

library(mlmRev)

library(ggeffects)

library(bbmle)

library(ggplot2)

library(lubridate)

library(DHARMa)

library(glmmTMB)

library(AICcmodavg)

library(MuMIn)

library(performance)

BatData <- read.csv("Lear_et_al_2023_BatAgaveData.csv",header=TRUE,sep=",")

BatData$reg <- factor(ifelse(BatData$region == 'Inf',1,0)) #create dummy variable for "region"

BatData$date <- as.Date(strptime(BatData$night, "%m/%d/%Y")) #format date; make "night" called "date"

BatData$doy <- yday(BatData$date) #format date; convert to day of year ("doy")

# TJP set output dir

out_dir <- 'C:\\Users\\klrat\\Desktop\\output'

######################################################################

###Run summary statistics on the variables###

######################################################################

BatData$bathrs <- BatData$num_bats*(BatData$minutes/60)

BatData$vpbph <- BatData$visits/BatData$bathrs #visits per bat per hour

BatData[BatData$visits == 0 & BatData$bathrs == 0,'vpbph']=0

BatData$vph <- BatData$visits/(BatData$minutes/60)

#make a table of variable ranges

tabvars <- c("visits","vpbph","vph","vpm","flybys","fbpm","elev",

"slope","tot_umbs","tot_grps","open_umbs","UmbPerTot","open_grps" , "GrpPerTot" ,

"relposmid", "relpostop", "dens_stlk" , "dens_emer" , "dens_young" ,"dens_open" ,"dens_fuzz" , "dens_dry" , "num_bats")

vtab <- data.frame(param = tabvars)

vtab$min <- round(sapply(tabvars, function(x) min(BatData[,x])),4)

vtab$max <- round(sapply(tabvars, function(x) max(BatData[,x])),4)

vtab$mean <- round(sapply(tabvars, function(x) mean(BatData[,x])),4)

vtab$sd <- round(sapply(tabvars, function(x) sd(BatData[,x])),4)

vtab$region <- "combined"

rocvtab <- data.frame(param = tabvars)

rocvtab$min <- round(sapply(tabvars, function(x) min(BatData[BatData$region == 'Ros',x])),4)

rocvtab$max <- round(sapply(tabvars, function(x) max(BatData[BatData$region == 'Ros',x])),4)

rocvtab$mean <- round(sapply(tabvars, function(x) mean(BatData[BatData$region == 'Ros',x])),4)

rocvtab$sd <- round(sapply(tabvars, function(x) sd(BatData[BatData$region == 'Ros',x])),4)

rocvtab$region <- "Roc"

infvtab <- data.frame(param = tabvars)

infvtab$min <- round(sapply(tabvars, function(x) min(BatData[BatData$region == 'Inf',x])),4)

infvtab$max <- round(sapply(tabvars, function(x) max(BatData[BatData$region == 'Inf',x])),4)

infvtab$mean <- round(sapply(tabvars, function(x) mean(BatData[BatData$region == 'Inf',x])),4)

infvtab$sd <- round(sapply(tabvars, function(x) sd(BatData[BatData$region == 'Inf',x])),4)

infvtab$region <- "Inf"

vtab <- rbind(vtab,rocvtab)

vtab <- rbind(vtab, infvtab)

vtab

write.csv(vtab, file.path(out_dir, "variable_summary.csv"), row.names = F)

#-----Calculate if any bats were present in a cluster on a given night, based on if any flybys happened at any focal agave in the cluster.

BatData$uclus <- paste(BatData$night,BatData$cluster,sep = "_")

BatData$anypres <- NA

auclus <- unique(BatData$uclus)

for(i in 1:length(auclus)){

tflyby <- BatData[BatData$uclus == auclus[i],'flybys']

mxflyby <- ifelse(max(tflyby)>0,1,0)

BatData[BatData$uclus == auclus[i],'anypres']=mxflyby

}

BatData$anypres <- factor(BatData$anypres)

BatData$present <- factor(ifelse(BatData$flybys > 0,1,0))

options(max.print=10000)

BatData

######################################################################

###Create standardized data set###

######################################################################

#package "glmmTMB" info"

#From: Bolker March 15, 2020: https://cran.r-project.org/web/packages/glmmTMB/vignettes/glmmTMB.pdf

#From: Brooks et al. 2017: https://journal.r-project.org/archive/2017/RJ-2017-066/RJ-2017-066.pdf

#https://cran.r-project.org/web/packages/glmmTMB/glmmTMB.pdf

#https://journal.r-project.org/archive/2017/RJ-2017-066/index.html

theme_set(theme_bw()+theme(panel.spacing=grid::unit(0,"lines"))) #cosmetic

#-----Create a standardized dataset using a z-score transformation. Each variable will now have a mean of 0 and an SD of 1.

sdat <- BatData[,c('visits','reg','site','cluster','FINAL_ID','anypres',"minutes","num_bats")] #these are the non-transformed variables

mvars <- c("slope" , "tot_grps", "open_grps", "relposmid", "dens_stlk", "dens_emer",

"dens_young","dens_open", "dens_fuzz", "dens_dry","open_umbs","tot_umbs", "elev")

mvar_mn <- c()

mvar_sd <- c()

for(m in mvars){

mvar_mn <- c(mvar_mn,mean(BatData[,m]))

mvar_sd <- c(mvar_sd,sd(BatData[,m]))

sdat[,m]=(BatData[,m]-mean(BatData[,m]))/sd(BatData[,m])

}

sdat$myoffset <- (sdat$num_bats*sdat$minutes)+1 #create offset

uclus <- factor(BatData$uclus)

sdat <- cbind(sdat, uclus) #add "uclus" to input data file; will use as random effect in zero-inflation component of models

######################################################################

###Build candidate model set###

######################################################################

modFullRegZIP0 <- glmmTMB(visits ~ reg + slope + tot_umbs + open_umbs + relposmid +

dens_open + dens_fuzz + dens_dry + open_umbs*relposmid + I(relposmid^2) + (1|site/cluster/FINAL_ID) +

offset(log(myoffset)), zi = ~(1|uclus), family = poisson, data = sdat)

modFullZIP0 <- glmmTMB(visits ~ slope + tot_umbs + open_umbs + relposmid +

dens_open + dens_fuzz + dens_dry + open_umbs*relposmid + I(relposmid^2) + (1|site/cluster/FINAL_ID) +

offset(log(myoffset)), zi = ~(1|uclus), family = poisson, data = sdat)

modNullZIP0 <- glmmTMB(visits ~ 1 + (1|site/cluster/FINAL_ID) +

offset(log(myoffset)), zi = ~(1|uclus), family = poisson, data = sdat)

modNoIntsRegZIP0 <- glmmTMB(visits ~ reg + slope + tot_umbs + open_umbs + relposmid +

dens_open + dens_fuzz + dens_dry + I(relposmid^2) + (1|site/cluster/FINAL_ID) +

offset(log(myoffset)), zi = ~(1|uclus), family = poisson, data = sdat)

modNoIntsZIP0 <- glmmTMB(visits ~ slope + tot_umbs + open_umbs + relposmid +

dens_open + dens_fuzz + dens_dry + I(relposmid^2) + (1|site/cluster/FINAL_ID) +

offset(log(myoffset)), zi = ~(1|uclus), family = poisson, data = sdat)

modNoQuadsRegZIP0 <- glmmTMB(visits ~ reg + slope + tot_umbs + open_umbs + relposmid +

dens_open + dens_fuzz + dens_dry + open_umbs*relposmid + (1|site/cluster/FINAL_ID) +

offset(log(myoffset)), zi = ~(1|uclus), family = poisson, data = sdat)

modNoQuadsZIP0 <- glmmTMB(visits ~ slope + tot_umbs + open_umbs + relposmid +

dens_open + dens_fuzz + dens_dry + open_umbs*relposmid + (1|site/cluster/FINAL_ID) +

offset(log(myoffset)), zi = ~(1|uclus), family = poisson, data = sdat)

modLinRegZIP0 <- glmmTMB(visits ~ reg + slope + tot_umbs + open_umbs + relposmid +

dens_open + dens_fuzz + dens_dry + (1|site/cluster/FINAL_ID) +

offset(log(myoffset)), zi = ~(1|uclus), family = poisson, data = sdat)

modLinZIP0 <- glmmTMB(visits ~ slope + tot_umbs + open_umbs + relposmid +

dens_open + dens_fuzz + dens_dry + (1|site/cluster/FINAL_ID) +

offset(log(myoffset)), zi = ~(1|uclus), family = poisson, data = sdat)

modAgaveRegZIP0 <- glmmTMB(visits ~ reg + tot_umbs + open_umbs + relposmid + open_umbs*relposmid + I(relposmid^2) + (1|site/cluster/FINAL_ID) +

offset(log(myoffset)), zi = ~(1|uclus), family = poisson, data = sdat)

modAgaveZIP0 <- glmmTMB(visits ~ tot_umbs + open_umbs + relposmid + open_umbs*relposmid + I(relposmid^2) + (1|site/cluster/FINAL_ID) +

offset(log(myoffset)), zi = ~(1|uclus), family = poisson, data = sdat)

modPatchRegZIP0 <- glmmTMB(visits ~ reg + slope + dens_open + dens_fuzz + dens_dry +

(1|site/cluster/FINAL_ID) +

offset(log(myoffset)), zi = ~(1|uclus), family = poisson, data = sdat)

modPatchZIP0 <- glmmTMB(visits ~ slope + dens_open + dens_fuzz + dens_dry + (1|site/cluster/FINAL_ID) +

offset(log(myoffset)), zi = ~(1|uclus), family = poisson, data = sdat)

modOber1ZIP0 <- glmmTMB(visits ~ open_umbs + relposmid + open_umbs*relposmid + I(relposmid^2) + (1|site/cluster/FINAL_ID) +

offset(log(myoffset)), zi = ~(1|uclus), family = poisson, data = sdat)

modOber2ZIP0 <- glmmTMB(visits ~ open_umbs + relposmid + (1|site/cluster/FINAL_ID) +

offset(log(myoffset)), zi = ~(1|uclus), family = poisson, data = sdat)

modOber3ZIP0 <- glmmTMB(visits ~ open_umbs + relposmid + I(relposmid^2) + (1|site/cluster/FINAL_ID) +

offset(log(myoffset)), zi = ~(1|uclus), family = poisson, data = sdat)

modOber4ZIP0 <- glmmTMB(visits ~ open_umbs + relposmid + open_umbs*relposmid + (1|site/cluster/FINAL_ID) +

offset(log(myoffset)), zi = ~(1|uclus), family = poisson, data = sdat)

modOber1RegZIP0 <- glmmTMB(visits ~ reg + open_umbs + relposmid + open_umbs*relposmid + I(relposmid^2) + (1|site/cluster/FINAL_ID) +

offset(log(myoffset)), zi = ~(1|uclus), family = poisson, data = sdat)

modOber2RegZIP0 <- glmmTMB(visits ~ reg + open_umbs + relposmid + (1|site/cluster/FINAL_ID) +

offset(log(myoffset)), zi = ~(1|uclus), family = poisson, data = sdat)

modOber3RegZIP0 <- glmmTMB(visits ~ reg + open_umbs + relposmid + I(relposmid^2) + (1|site/cluster/FINAL_ID) +

offset(log(myoffset)), zi = ~(1|uclus), family = poisson, data = sdat)

modOber4RegZIP0 <- glmmTMB(visits ~ reg + open_umbs + relposmid + open_umbs*relposmid + (1|site/cluster/FINAL_ID) +

offset(log(myoffset)), zi = ~(1|uclus), family = poisson, data = sdat)

######################################################################

###Examine model results###

######################################################################

#-----AICc of all models:

modnamesZIP <- c('modFullRegZIP0', 'modFullZIP0', 'modNullZIP0', 'modNoIntsRegZIP0', 'modNoIntsZIP0', 'modNoQuadsRegZIP0', 'modNoQuadsZIP0',

'modLinRegZIP0', 'modLinZIP0', 'modAgaveRegZIP0', 'modAgaveZIP0', 'modPatchRegZIP0',

'modPatchZIP0', 'modOber1ZIP0', 'modOber2ZIP0', 'modOber3ZIP0', 'modOber4ZIP0', 'modOber1RegZIP0',

'modOber2RegZIP0', 'modOber3RegZIP0', 'modOber4RegZIP0')

amodsZIP <- list(modFullRegZIP0, modFullZIP0, modNullZIP0, modNoIntsRegZIP0, modNoIntsZIP0, modNoQuadsRegZIP0, modNoQuadsZIP0,

modLinRegZIP0, modLinZIP0, modAgaveRegZIP0, modAgaveZIP0, modPatchRegZIP0,

modPatchZIP0, modOber1ZIP0, modOber2ZIP0, modOber3ZIP0, modOber4ZIP0, modOber1RegZIP0,

modOber2RegZIP0, modOber3RegZIP0, modOber4RegZIP0)

atabZIP <- aictab(amodsZIP, modnames = modnamesZIP, second.ord = TRUE, nobs = NULL, sort = TRUE, c.hat = 1) #with proper format to use aiccmodavg package

write.table(atabZIP, file=file.path(out_dir, "ModelTable.csv"), sep=",")

#-----Get R-squared values for each model:

#gives performance stats on all models, including conditional and marginal R2 for each model:

performance_table <- compare_performance(modFullRegZIP0, modFullZIP0, modNullZIP0, modNoIntsRegZIP0, modNoIntsZIP0, modNoQuadsRegZIP0, modNoQuadsZIP0,

modLinRegZIP0, modLinZIP0, modAgaveRegZIP0, modAgaveZIP0, modPatchRegZIP0,

modPatchZIP0, modOber1ZIP0, modOber2ZIP0, modOber3ZIP0, modOber4ZIP0, modOber1RegZIP0,

modOber2RegZIP0, modOber3RegZIP0, modOber4RegZIP0, rank = TRUE)

write.table(performance_table, file=file.path("PerformanceTable.csv"), sep=",")

######################################################################

###Make confidence set of models using the level of 0.95###

######################################################################

cset = confset(cand.set=amodsZIP, modnames=modnamesZIP, level=0.95, c.hat = 1)

csetnames <- as.character(cset$table$Modnames)

######################################################################

###Make a table of the parameter estimates in the models of the confidence set###

######################################################################

ptab <- data.frame()

for(i in 1:length(csetnames)){

ttab <- as.data.frame(summary(get(csetnames[i]))$coefficients$cond)

ttab$coef <- row.names(ttab)

ttab$mod <- csetnames[i]

ptab <- rbind(ptab,ttab)

}

sigpars <- sort(unique(ptab[ptab$`Pr(>|z|)`< 0.05,'coef'])) #all significant parameters at p < 0.05 level (from any model in cset)

sigpars_noint <- sigpars[(sapply(sigpars,function(x) !grepl(":",x, fixed = TRUE))) & sigpars!="(Intercept)"]

write.table(ptab, file=file.path(out_dir,"CoefficientTable.csv"), sep=",")

######################################################################

###Model average the parameter estimates using the confidence set and standard errors and create a table of these values###

######################################################################

modnamesAVG <- c('modNoQuadsZIP0', 'modNoQuadsRegZIP0', 'modFullZIP0', 'modLinZIP0', 'modOber2RegZIP0', 'modOber2ZIP0', 'modNoIntsZIP0', 'modFullRegZIP0',

'modLinRegZIP0', 'modOber4RegZIP0', 'modOber3RegZIP0', 'modOber4ZIP0', 'modOber3ZIP0')

amodsAVG <- list(modNoQuadsZIP0, modNoQuadsRegZIP0, modFullZIP0, modLinZIP0, modOber2RegZIP0, modOber2ZIP0, modNoIntsZIP0, modFullRegZIP0,

modLinRegZIP0, modOber4RegZIP0, modOber3RegZIP0, modOber4ZIP0, modOber3ZIP0)

modelavgparamests <- model.avg(amodsAVG)

modelavgparamestsTable <- coefTable(modelavgparamests, full = TRUE, se = TRUE) #use the "full" averaging method which uses the "zero method" for calculating average parameter estimates

write.table(modelavgparamestsTable, file=file.path(out_dir, "ModelAveragedParameterEstimates.csv"), sep=",")

######################################################################

###Define variable names###

######################################################################

var_names = list("open_umbs" = "Number of umbels with\n open flowers (open_umbs)",

"relposmid" = "Relative vertical position of umbels\n with open flowers (relposmid)",

"dens_open" = "Density of agaves with\n open flowers (dens_open)",

"dens_dry" = "Density of dead standing\n stalks (dens_dry)")

######################################################################

###Model average the 95% confidence set of models to get model-averaged predictions###

######################################################################

single_col_in = 3.14961

double_col_in = 6.29921

plot_res = 800

#-----Make a new dataset for prediction holding all variables constant at mean (except for variable of interest)

pname <- 'dens_dry'

pvar <- which(names(sdat) == pname)

mind <- which(mvars == pname)

pvals <- seq(min(sdat[,pvar]),max(sdat[,pvar]),length.out = 100)

pdat <- as.data.frame(pvals)

othvars <- mvars[!(mvars==pvar)]

for(i in othvars){

pdat[,i]=0.0

}

names(pdat)[1] <- pname

pdat$anypres = 0

pdat$reg <- 1

pdat$site <- NA

pdat$cluster <- NA

pdat$FINAL_ID <- NA

pdat$uclus <- NA

pdat$myoffset <- (1*60)+1 #set offset equivalent to 1 bat and 60 minutes

pdat$realval <- (pdat[,pname]*mvar_sd[mind])+mvar_mn[mind] #undo z-score (back to real values)

#-----Get predictions for each model in cset

mcset <- lapply(csetnames,function(x) get(x))

avgm <- model.avg(mcset)

avgp <- predict(avgm, pdat, se.fit = T,type = 'link')

pdat$p_mn <- exp(avgp$fit)

pdat$p_lse <- exp(avgp$fit-avgp$se.fit)

pdat$p_use <- exp(avgp$fit+avgp$se.fit)

ilink <- family(amodsZIP[[1]])$linkinv #get the inverse link function

pdat$p_lci <- ilink(avgp$fit - (1.96 * avgp$se.fit)) #lower 95% CI

pdat$p_uci <- ilink(avgp$fit + (1.96 * avgp$se.fit)) #upper 95% CI

#-----Make plots

plot_lab <- as.character(var_names[pname])

pdat <- pdat[,c('realval','p_mn','p_lci','p_uci','p_lse','p_use')]

#make plot with confidence intervals

x11(width = single_col_in, height = single_col_in) #make a graph the exact size you want

# par(ps = 10) #, mai = c(.75,.75,.25,.1), mgp = c(1.6,.5,0))

plt <- ggplot(pdat, aes(x = realval, y = p_mn)) +

geom_line() +

labs(x = plot_lab, y = 'Predicted visits per bat per hour') +

theme(panel.grid.major = element_blank(), panel.grid.minor = element_blank()) +

theme(axis.title = element_text(face = "plain"),

text = element_text(size=10))

plt + geom_ribbon(data = pdat,

aes(ymin = p_lci, ymax = p_uci),

alpha = 0.1)

out_name <- paste0("predplot_ribbon_", pname, ".tif")

ggsave(

filename = out_name,

device = "tiff",

path = out_dir,

width = single_col_in,

height = single_col_in,

dpi = 800

)

print(paste("prediction at: ",pdat$realval[1], " ", pname, " = ", pdat$p_mn[1], " visits"))

print(paste("prediction at: ",pdat$realval[nrow(pdat)], " ", pname, " = ", pdat$p_mn[nrow(pdat)], " visits"))

# #make plot with standard errors

# x11(width = 4, height = 4) #make a graph the exact size you want

# par(ps = 11) #, mai = c(.75,.75,.25,.1), mgp = c(1.6,.5,0))

# plt <- ggplot(pdat, aes(x = realval, y = p_mn)) +

# geom_line() +

# labs(x = pname, y = 'Predicted Visits per Bat per Hour')

# plt + geom_ribbon(data = pdat,

# aes(ymin = p_lse, ymax = p_use),

# alpha = 0.1)

#-----Make plot of open_umbs*relposmid interaction

pname <- "open_umbs"

pvar <- which(names(sdat) == pname)

mind <- which(mvars == pname)

pvals <- seq(min(sdat[,pvar]),max(sdat[,pvar]),length.out = 100)

pdat <- as.data.frame(pvals)

othvars <- mvars[!(mvars==pvar)]

for(i in othvars){

pdat[,i]=0.0

}

names(pdat)[1] <- pname

pdat$anypres = 0

pdat$reg <- 1

pdat$site <- NA

pdat$cluster <- NA

pdat$FINAL_ID <- NA

pdat$uclus <- NA

pdat$myoffset <- (1*60)+1 #set offset equivalent to 1 bat and 60 minutes

pdat$realval <- (pdat[,pname]*mvar_sd[mind])+mvar_mn[mind] #undo z-score (back to real values)

#get predictions with relposmid set to 1SD lower than mean

pdat$relposmid <- -1.0

lowpred <- predict(avgm, pdat, se.fit = T,type = 'link',backtransform = F)

pdat$plow <- ilink(lowpred$fit)

pdat$plow_lci <- ilink(lowpred$fit - (1.96 * lowpred$se.fit)) #lower 95% CI

pdat$plow_uci <- ilink(lowpred$fit + (1.96 * lowpred$se.fit)) #lower 95% CI

print(paste("prediction at: ",pdat$realval[1], " ", pname, " = ", pdat$plow[1], " visits for relposmid=",pdat$relposmid[1]))

print(paste("prediction at: ",pdat$realval[nrow(pdat)], " ", pname, " = ", pdat$plow[nrow(pdat)], " visits for relposmid=",pdat$relposmid[1]),"SD")

#get predictions with relposmid set to 1SD higher than mean

pdat$relposmid <- 1.0

highpred <- predict(avgm, pdat, se.fit = T,type = 'link',backtransform = F)

pdat$phigh <- ilink(highpred$fit)

pdat$phigh_lci <- ilink(highpred$fit - (1.96 * highpred$se.fit)) #lower 95% CI

pdat$phigh_uci <- ilink(highpred$fit + (1.96 * highpred$se.fit)) #lower 95% CI

print(paste("prediction at: ",pdat$realval[1], " ", pname, " = ", pdat$phigh[1], " visits for relposmid=",pdat$relposmid[1]))

print(paste("prediction at: ",pdat$realval[nrow(pdat)], " ", pname, " = ", pdat$phigh[nrow(pdat)], " visits for relposmid=",pdat$relposmid[1]),"SD")

#get predictions with relposmid set to mean

pdat$relposmid <- 0.0

mnpred <- predict(avgm, pdat, se.fit = T,type = 'link',backtransform = F)

pdat$pmean <- ilink(mnpred$fit)

pdat$pmean_lci <- ilink(mnpred$fit - (1.96 * mnpred$se.fit)) #lower 95% CI

pdat$pmean_uci <- ilink(mnpred$fit + (1.96 * mnpred$se.fit)) #lower 95% CI

print(paste("prediction at: ",pdat$realval[1], " ", pname, " = ", pdat$pmean[1], " visits for relposmid=",pdat$relposmid[1]))

print(paste("prediction at: ",pdat$realval[nrow(pdat)], " ", pname, " = ", pdat$pmean[nrow(pdat)], " visits for relposmid=",pdat$relposmid[1]),"SD")

#get actual values of relposmid standard deviations

sdvals <- round(sapply(c(-1,0,1), function(x) x*sd(BatData$relposmid)+mean(BatData$relposmid)),2)

pdat <- pdat[,c('realval','plow','plow_lci','plow_uci','phigh','phigh_lci','phigh_uci',

'pmean','pmean_lci','pmean_uci')]

pdat <- pdat[pdat$realval <= 20,]

yrange <- c(0,200)

xrange <- range(pdat$realval)

#Make plots with consistent y-axis

x11(width = double_col_in, height = 4)

par(mfrow = c(1,3), ps = 9, mai = c(.75,.75,.25,.1), mgp = c(1.6,.5,0),cex = 1)

ylab_text = "Predicted visits per bat per hour"

plot(0,type = 'n',ylim = yrange,xlim = xrange,ylab = ylab_text, xlab = pname,

main = paste("relposmid =",sdvals[1]), font.main = 1)

polygon(c(pdat$realval,rev(pdat$realval)),c(pdat$plow_lci,rev(pdat$plow_uci)),col = 'grey80',border=NA)

lines(pdat$realval,pdat$plow,lwd = 2)

plot(0,type = 'n',ylim = yrange,xlim = xrange,ylab = ylab_text, xlab = pname,

main = paste("relposmid =",sdvals[2]), font.main = 1)

polygon(c(pdat$realval,rev(pdat$realval)),c(pdat$pmean_lci,rev(pdat$pmean_uci)),col = 'grey80',border=NA)

lines(pdat$realval,pdat$pmean,lwd = 2)

plot(0,type = 'n',ylim = yrange,xlim = xrange,ylab = ylab_text, xlab = pname,

main = paste("relposmid =",sdvals[3]), font.main = 1)

polygon(c(pdat$realval,rev(pdat$realval)),c(pdat$phigh_lci,rev(pdat$phigh_uci)),col = 'grey80',border=NA)

lines(pdat$realval,pdat$phigh,lwd = 2)

ofilenm <- file.path(out_dir, paste0("presp_umb_x_relpos", ".tif"))

#ofilenm <- paste(wdir,"//","figures//","presp_umb_x_relpos.jpg",sep = '')

dev.print(tiff, file = ofilenm, width = double_col_in, height = 4, res = 800, units = "in") #use this to save

#Make plots with variable y-axis

par(mfrow = c(1,3), ps = 9, mai = c(.75,.75,.25,.1), mgp = c(1.6,.5,0),cex = 1)

plot(0,type = 'n',ylim = range(c(0,max(pdat$plow_uci))),xlim = xrange,ylab = "Predicted Visits per Bat per Hour", xlab = pname,

main = paste("relposmid =",sdvals[1]))

polygon(c(pdat$realval,rev(pdat$realval)),c(pdat$plow_lci,rev(pdat$plow_uci)),col = 'grey80',border=NA)

lines(pdat$realval,pdat$plow,lwd = 2)

plot(0,type = 'n',ylim = range(c(0,max(pdat$pmean_uci))),xlim = xrange,ylab = "Predicted Visits per Bat per Hour", xlab = pname,

main = paste("relposmid =",sdvals[2]))

polygon(c(pdat$realval,rev(pdat$realval)),c(pdat$pmean_lci,rev(pdat$pmean_uci)),col = 'grey80',border=NA)

lines(pdat$realval,pdat$pmean,lwd = 2)

plot(0,type = 'n',ylim = range(c(0,max(pdat$phigh_uci))),xlim = xrange,ylab = "Predicted Visits per Bat per Hour", xlab = pname,

main = paste("relposmid =",sdvals[3]))

polygon(c(pdat$realval,rev(pdat$realval)),c(pdat$phigh_lci,rev(pdat$phigh_uci)),col = 'grey80',border=NA)

lines(pdat$realval,pdat$phigh,lwd = 2)

#-------------------------Make plots with ggplot

# library(ggplot2)

# library(tidyr)

# library(dplyr)

#

# # Reshape the data into a long format for the predicted values

# long_data <- pdat %>%

# pivot_longer(cols = c("plow", "phigh", "pmean"), names_to = "type", values_to = "value")

#

# # Reshape the data into a long format for the confidence intervals

# long_lci <- pdat %>%

# pivot_longer(cols = c("plow_lci", "phigh_lci", "pmean_lci"), names_to = "type", values_to = "lci") %>%

# mutate(type = sub("_lci", "", type))

#

# long_uci <- pdat %>%

# pivot_longer(cols = c("plow_uci", "phigh_uci", "pmean_uci"), names_to = "type", values_to = "uci") %>%

# mutate(type = sub("_uci", "", type))

#

# # Join the confidence interval data back to the main data frame

# long_data <- long_data %>%

# left_join(long_lci, by = c("realval", "type")) %>%

# left_join(long_uci, by = c("realval", "type"))

#

# long_data$type <- factor(long_data$type, levels = c('plow', 'pmean', 'phigh'))

#

# # Now create the ggplot with facets

# gg <- ggplot(long_data, aes(x = realval, y = value)) +

# geom_ribbon(aes(ymin = lci, ymax = uci, fill = type), alpha = 0.3) +

# geom_line(aes(color = type), size = 1) +

# facet_wrap(~ type, ncol = 3) +

# scale_fill_manual(values = c("grey80", "grey80", "grey80")) +

# scale_color_manual(values = c("black", "black", "black")) +

# scale_y_continuous(limits = c(0, 200)) +

# labs(x = "Predicted Visits",

# y = "Visits per Bat per Hour") +

# theme_minimal() +

# theme(legend.position = "none",

# #strip.background = element_blank(),

# # axis.title.y = element_blank(),

# # axis.text.y = element_blank(),

# # axis.ticks.y = element_blank(),

# axis.title.x = element_text(margin = margin(t = 10)))

#

# # Print the plot

# print(gg)

#-------------------------

#-----Make plot of open_umbs*relposmid interaction with the axes reversed

pname <- "relposmid"

pvar <- which(names(sdat) == pname)

mind <- which(mvars == pname)

pvals <- seq(min(sdat[,pvar]),max(sdat[,pvar]),length.out = 100)

pdat <- as.data.frame(pvals)

othvars <- mvars[!(mvars==pvar)]

for(i in othvars){

pdat[,i]=0.0

}

names(pdat)[1] <- pname

pdat$anypres = 0

pdat$reg <- 1

pdat$site <- NA

pdat$cluster <- NA

pdat$FINAL_ID <- NA

pdat$uclus <- NA

pdat$myoffset <- (1*60)+1 #set offset equivalent to 1 bat and 60 minutes

pdat$realval <- (pdat[,pname]*mvar_sd[mind])+mvar_mn[mind] #undo z-score (back to real values)

#get predictions with open_umbs set to 1SD lower than mean

pdat$open_umbs <- -1.0

lowpred <- predict(avgm, pdat, se.fit = T,type = 'link',backtransform = F)

pdat$plow <- ilink(lowpred$fit)

pdat$plow_lci <- ilink(lowpred$fit - (1.96 * lowpred$se.fit)) #lower 95% CI

pdat$plow_uci <- ilink(lowpred$fit + (1.96 * lowpred$se.fit)) #lower 95% CI

print(paste("prediction at: ",pdat$realval[1], " ", pname, " = ", pdat$plow[1], " visits for open_umbs=",pdat$open_umbs[1],"SD"))

print(paste("prediction at: ",pdat$realval[nrow(pdat)], " ", pname, " = ", pdat$plow[nrow(pdat)], " visits for open_umbs=",pdat$open_umbs[1],"SD"))

#get predictions with open_umbs set to 1SD higher than mean

pdat$open_umbs <- 1.0

highpred <- predict(avgm, pdat, se.fit = T,type = 'link',backtransform = F)

pdat$phigh <- ilink(highpred$fit)

pdat$phigh_lci <- ilink(highpred$fit - (1.96 * highpred$se.fit)) #lower 95% CI

pdat$phigh_uci <- ilink(highpred$fit + (1.96 * highpred$se.fit)) #lower 95% CI

print(paste("prediction at: ",pdat$realval[1], " ", pname, " = ", pdat$phigh[1], " visits for open_umbs=",pdat$open_umbs[1],"SD"))

print(paste("prediction at: ",pdat$realval[nrow(pdat)], " ", pname, " = ", pdat$phigh[nrow(pdat)], " visits for open_umbs=",pdat$open_umbs[1],"SD"))

#get predictions with open_umbs set to mean

pdat$open_umbs <- 0.0

mnpred <- predict(avgm, pdat, se.fit = T,type = 'link',backtransform = F)

pdat$pmean <- ilink(mnpred$fit)

pdat$pmean_lci <- ilink(mnpred$fit - (1.96 * mnpred$se.fit)) #lower 95% CI

pdat$pmean_uci <- ilink(mnpred$fit + (1.96 * mnpred$se.fit)) #lower 95% CI

print(paste("prediction at: ",pdat$realval[1], " ", pname, " = ", pdat$pmean[1], " visits for open_umbs=",pdat$open_umbs[1],"SD"))

print(paste("prediction at: ",pdat$realval[nrow(pdat)], " ", pname, " = ", pdat$pmean[nrow(pdat)], " visits for open_umbs=",pdat$open_umbs[1],"SD"))

#get actual values of open_umbs standard deviations

sdvals <- round(sapply(c(-1,0,1), function(x) x*sd(BatData$open_umbs)+mean(BatData$open_umbs)),2)

pdat <- pdat[,c('realval','plow','plow_lci','plow_uci','phigh','phigh_lci','phigh_uci',

'pmean','pmean_lci','pmean_uci')]

yrange <- c(0,200)

xrange <- range(pdat$realval)

#Make plots with consistent y-axis

x11(width = double_col_in, height = 4)

par(mfrow = c(1,3), ps = 9, mai = c(.75,.75,.25,.1), mgp = c(1.6,.5,0),cex = 1)

plot(0,type = 'n',ylim = yrange,xlim = xrange,ylab = ylab_text, xlab = pname,

main = paste("open_umbs =",sdvals[1]), font.main = 1)

polygon(c(pdat$realval,rev(pdat$realval)),c(pdat$plow_lci,rev(pdat$plow_uci)),col = 'grey80',border=NA)

lines(pdat$realval,pdat$plow,lwd = 2)

plot(0,type = 'n',ylim = yrange,xlim = xrange,ylab = ylab_text, xlab = pname,

main = paste("open_umbs =",sdvals[2]), font.main = 1)

polygon(c(pdat$realval,rev(pdat$realval)),c(pdat$pmean_lci,rev(pdat$pmean_uci)),col = 'grey80',border=NA)

lines(pdat$realval,pdat$pmean,lwd = 2)

plot(0,type = 'n',ylim = yrange,xlim = xrange,ylab = ylab_text, xlab = pname,

main = paste("open_umbs =",sdvals[3]), font.main = 1)

polygon(c(pdat$realval,rev(pdat$realval)),c(pdat$phigh_lci,rev(pdat$phigh_uci)),col = 'grey80',border=NA)

lines(pdat$realval,pdat$phigh,lwd = 2)

ofilenm <- file.path(out_dir, paste0("presp_relposxopenumb", ".tif"))

dev.print(tiff, file = ofilenm, width = double_col_in, height = 4, res = 800, units = "in") #use this to save

############################End of Code################################
